# Role of the B Allele of Influenza A Virus Segment 8 in Setting Mammalian Host Range and Pathogenicity

**DOI:** 10.1128/JVI.01205-16

**Published:** 2016-09-29

**Authors:** Matthew L. Turnbull, Helen M. Wise, Marlynne Q. Nicol, Nikki Smith, Rebecca L. Dunfee, Philippa M. Beard, Brett W. Jagger, Yvonne Ligertwood, Gareth R. Hardisty, Haixia Xiao, Donald J. Benton, Alice M. Coburn, Joao A. Paulo, Steven P. Gygi, John W. McCauley, Jeffery K. Taubenberger, Samantha J. Lycett, Michael P. Weekes, Bernadette M. Dutia, Paul Digard

**Affiliations:** a Division of Infection and Immunity, The Roslin Institute, The University of Edinburgh, Easter Bush, Midlothian, Edinburgh, United Kingdom; b Viral Pathogenesis and Evolution Section, Laboratory of Infectious Diseases, Division of Intramural Research, National Institute of Allergy and Infectious Diseases, Bethesda, Maryland, USA; c Department of Pathology, Division of Virology, University of Cambridge, Cambridge, United Kingdom; d The Francis Crick Institute, Mill Hill Laboratory, Mill Hill, London, United Kingdom; e The Centre for Virus Research, The University of Glasgow, Glasgow, United Kingdom; f Department of Cell Biology, Harvard Medical School, Boston, Massachusetts, USA; g Cambridge Institute for Medical Research, University of Cambridge, Cambridge, United Kingdom; Icahn School of Medicine at Mount Sinai

## Abstract

Two alleles of segment 8 (NS) circulate in nonchiropteran influenza A viruses. The A allele is found in avian and mammalian viruses, but the B allele is viewed as being almost exclusively found in avian viruses. This might reflect the fact that one or both of its encoded proteins (NS1 and NEP) are maladapted for replication in mammalian hosts. To test this, a number of clade A and B avian virus-derived NS segments were introduced into human H1N1 and H3N2 viruses. In no case was the peak virus titer substantially reduced following infection of various mammalian cell types. Exemplar reassortant viruses also replicated to similar titers in mice, although mice infected with viruses with the avian virus-derived segment 8s had reduced weight loss compared to that achieved in mice infected with the A/Puerto Rico/8/1934 (H1N1) parent. *In vitro*, the viruses coped similarly with type I interferons. Temporal proteomics analysis of cellular responses to infection showed that the avian virus-derived NS segments provoked lower levels of expression of interferon-stimulated genes in cells than wild type-derived NS segments. Thus, neither the A nor the B allele of avian virus-derived NS segments necessarily attenuates virus replication in a mammalian host, although the alleles can attenuate disease. Phylogenetic analyses identified 32 independent incursions of an avian virus-derived A allele into mammals, whereas 6 introductions of a B allele were identified. However, A-allele isolates from birds outnumbered B-allele isolates, and the relative rates of Aves-to-Mammalia transmission were not significantly different. We conclude that while the introduction of an avian virus segment 8 into mammals is a relatively rare event, the dogma of the B allele being especially restricted is misleading, with implications in the assessment of the pandemic potential of avian influenza viruses.

**IMPORTANCE** Influenza A virus (IAV) can adapt to poultry and mammalian species, inflicting a great socioeconomic burden on farming and health care sectors. Host adaptation likely involves multiple viral factors. Here, we investigated the role of IAV segment 8. Segment 8 has evolved into two distinct clades: the A and B alleles. The B-allele genes have previously been suggested to be restricted to avian virus species. We introduced a selection of avian virus A- and B-allele segment 8s into human H1N1 and H3N2 virus backgrounds and found that these reassortant viruses were fully competent in mammalian host systems. We also analyzed the currently available public data on the segment 8 gene distribution and found surprisingly little evidence for specific avian host restriction of the B-clade segment. We conclude that B-allele segment 8 genes are, in fact, capable of supporting infection in mammals and that they should be considered during the assessment of the pandemic risk of zoonotic influenza A viruses.

## INTRODUCTION

Influenza A virus (IAV) belongs to the family Orthomyxoviridae and has a negative-sense RNA genome consisting of 8 single-stranded segments (1). IAV is subtyped according to its surface glycoproteins hemagglutinin (HA) and neuraminidase (NA), of which there are at least 16 and 9 different subtypes, respectively, in nonchiropteran strains. The natural host of IAV is waterfowl, but the virus is able to adapt to other avian and mammalian hosts. The virus causes seasonal epidemics and sporadic pandemics in humans, as well as regular outbreaks in wild and domestic animals. The determinants that facilitate the adaptation of an avian IAV to a new host species are incompletely understood at present. Host adaptation is likely influenced by a combination of multiple viral and host factors. Of the viral factors, the HA protein (2, 3), required for virus entry into host cells, and the polymerase basic protein 2 (PB2) (4), forming part of the trimeric RNA-dependent RNA polymerase, are thought to play particularly important roles in host adaptation, but most viral genes are likely to contribute (reviewed in reference 5). There is currently a global fear that avian influenza virus strains which are highly pathogenic in humans will adapt sufficiently to be able to spread readily within the human population. Thus, it is of high importance to improve our understanding of host adaptation and pathogenicity.

The nonstructural (NS) segment 8 of IAV encodes two major polypeptides that are expressed in all strains: nonstructural protein 1 (NS1) and the nuclear export protein (NEP). NS1 is expressed following faithful transcription of the segment 8 viral RNA (vRNA), while a pre-mRNA splicing event leads to NEP expression (6). IAV replicates its genome in the host cell nucleus, and NEP is essential for the nuclear export of viral ribonucleoproteins prior to virus egression (7). NEP has also been implicated in other roles, such as regulating viral genome replication (8) and assisting with virus budding (9). NS1 is a multifunctional, dimeric protein, ranging in size from 215 to 237 amino acids, that interacts with RNA and a plethora of host cell proteins in a strain- and host-specific manner to mediate its primary role of antagonizing the host innate immune response (reviewed in reference 10). The N-terminal 73 amino acids of NS1 constitute an RNA-binding domain (RBD) that can bind a variety of both single- and double-stranded RNAs with a low affinity (11, 12), and this is required to inhibit the host antiviral RNase L pathway by preventing activation of 2′-5′ oligoadenylate synthetase (OAS) (13). C terminal to the RBD, connected by a short linker, is an effector domain (ED) that forms interactions with many host cell factors. For example, the NS1 protein of many strains binds and inhibits the host 30-kDa cleavage and polyadenylation specificity factor (CPSF30) to inhibit host cell mRNA processing, thus dampening the innate immune response (14). The NS1 protein also binds host tripartite motif-containing protein 25 (TRIM25) and prevents retinoic acid-inducible gene I (RIG-I) ubiquitination following detection of pathogen-associated molecular markers (PAMPs) and subsequent activation, thus inhibiting the induction of the type I interferon (IFN) response (15). The products of the NS segment have indeed been implicated in host adaptation and pathogenicity. For instance, Li et al. concluded that the NS1 gene contributed to the virulence of H5N1 avian influenza viruses (16), while adaptive mutations in both the NS1 protein and NEP were found when A/Hong Kong/1968 (H3N2) was adapted to increased virulence in mice (17).

The NS segment of nonchiropteran IAV has classically been divided into two alleles on the basis of nucleotide homology, the A and B alleles (18), although recent findings from studies of H17 and H18 bat IAV isolates have initiated the use of the term C allele (19–21). There is a striking divergence in the amino acid homology of A- and B-allele NS1 proteins, where the identity between the lineages can be as low as 63% but is typically above 93% within an allele (22–24). The A- and B-allele groups encompass many avian IAV isolates, but historically, all strains isolated from mammalian hosts, bar one H3N8 equine IAV isolate (25), belonged to the A-allele group. Plausibly, this seeming bias reflects a host-specific restriction, and consistent with this, there is long-standing evidence that the B allele of the NS segment attenuates mammal-adapted viruses. Treanor et al. generated segment 8 reassortant viruses on the background of the human H3N2 A/Udorn/72 (Udorn72) virus and found that the B-allele reassortant gave significantly reduced peak titers in the nasopharynx of squirrel monkeys in comparison to an A-allele counterpart and also had a shorter duration of shedding (18). More recently, various B-allele NS1 proteins from avian viruses were found to be less effective than their A-allele counterparts at suppressing the activation of the beta IFN (IFN-β), NF-κB, and AP-1 promoters in human and mink lung cells (26–29). However, the introduction of a B-allele NS segment from an H5N1 highly pathogenic avian influenza (HPAI) virus into an HPAI H7N1 virus background increased virus fitness in mammalian cells and allowed productive infection of mice (30). Thus, there are conflicting data in the literature.

To better elucidate the role of the B-allele NS segment 8 in IAV host adaption, we introduced a variety of avian influenza virus A- and B-allele NS segments into human H1N1 viruses (laboratory-adapted or 2009 pandemic [pdm2009] viruses) and H3N2 viruses and assessed virus fitness in mammalian systems *in vitro* and *in vivo*. We found no evidence for a B-allele-specific attenuation of IAV replication or pathogenicity. We performed bioinformatic and phylogenetic analyses on all influenza virus segment 8 sequences available in GenBank and estimated the nominal rates of introduction of avian virus A- and B-allele NS segments into mammalian hosts. We propose that these rates are comparable and that the relative paucity of mammalian B-allele virus isolates reflects their lower prevalence in the avian host, a theory that has implications for the interpretation of surveillance data and assessment of pandemic risk (31).

## MATERIALS AND METHODS

### Ethics statement.

All animal experiments were carried out under the authority of a UK Home Office Project License (60/4479) within the terms and conditions of the strict regulations of the UK Home Office Animals (Scientific Procedures) Act 1986 and the code of practice for the housing and care of animals bred, supplied, or used for scientific purposes. Ethical approval for experiments using human macrophages was obtained from the Lothian Research Ethics Committee. All subjects provided written informed consent at each donation.

### Cell culture, viruses, plasmids, and antibodies.

Madin-Darby canine kidney (MDCK) cells, human embryonic kidney 293T cells (293T), and human adenocarcinoma alveolar basal epithelial (A549) cells (Sigma Co.) were grown in Dulbecco's modified Eagle medium (D-MEM; Sigma Co.) supplemented with 10% (vol/vol) fetal bovine serum (FBS), 2 mM glutamine, 100 U/ml penicillin, and 100 μg/ml streptomycin (complete D-MEM). Madin-Darby canine kidney sialyltransferase 1 (MDCK-SIAT) (32) cells were cultured as described above for MDCK cells with the addition of 1 mg/ml Geneticin (Gibco Co.) to the culture medium. HEK293-Blue IFN-α/β cells (HEK-Blue; InvivoGen) were cultured in complete D-MEM supplemented with 30 μg/ml blasticidin and 100 μg/ml zeocin (InvivoGen). Primary CD14^+^ human monocytes were isolated from whole blood as described previously (33). Monocytes were plated in 24-well plates in RPMI supplemented with 10% (vol/vol) FBS, 2 mM glutamine, 100 U/ml penicillin, 100 μg/ml streptomycin (Sigma Co.), and 10^4^ U/ml (100 ng/ml) recombinant human colony-stimulating factor 1 (rhCSF1; a gift from Chiron, Emeryville, CA, USA) for 7 days for differentiation into macrophages. Infections were performed on day 8.

A/Puerto Rico/8/1934 (H1N1) (PR8) (34), A/California/7/2009 (H1N1) (Cal7), and Udorn72 (H3N2) (35) viruses were generated by reverse genetics essentially as described previously (36). Briefly, 10^6^ human 293T cells were transfected with plasmids carrying viruses generated by reverse genetics (250 ng each), using 4 μl of a cationic lipid mixture (Lipofectamine 2000 transfection reagent; Invitrogen, Life Technologies) according to the manufacturer's instructions. To generate NS segment reassortants, the pDUAL plasmid for PR8 segment 8 was replaced with a pHH21 plasmid containing segment 8 from one of several low-pathogenic avian influenza (LPAI) virus strains (Table 1). These plasmids were constructed with full-length segment 8 cDNA generated by reverse transcription (RT)-PCR using LPAI virus RNA (37) and primers (38) following standard protocols (39). Additional pDUAL plasmids containing segment 8 cDNA from other relevant virus strains (Table 1) were generated by synthesizing full-length segment 8 genes (Biomatik) and cloning them into the pDUAL vector using BsmBI restriction endonuclease sites (primer sequences are available on request). Where full-length sequences, including the segment untranslated regions, were not available, segment 8 termini from the closest relative were used (Table 1). Any BsmBI DNA restriction endonuclease sites within the segment 8 cDNA sequence were synonymously changed to remove the site without altering the NS1 protein or NEP sequence (Table 1). Cal7 (H1N1) gene segments were amplified by RT-PCR (primer sequences are available on request) and cloned into the pHW2000 vector (40). On day 2, the medium was changed to serum-free D-MEM supplemented with 0.14% bovine serum albumin (wt/vol) and 5 μg/ml l-(tosylamido-2-phenyl) ethyl chloromethyl ketone (TPCK)-treated trypsin (Worthington Biochemicals) (RG medium). The supernatant was collected on day 4, and virus was propagated either by using 0.1 ml of virus-containing supernatant to infect T25 flasks of MDCK cells, maintained in 5 ml virus growth medium for 48 h, or by inoculating 2,000 PFU of virus into the allantoic cavity of 10-day-old embryonated chicken eggs that were maintained at 37°C for 72 h. Virus was titrated by standard plaque assays on MDCK or MDCK-SIAT (for Cal7-based viruses) cells. The sequence of segment 8 of each virus was verified.

**TABLE 1:** Virus strains

Virus name	Strain from which NS segment was isolated	Allele	GenBank accession no. (NS segment)
PR8	A/Puerto Rico/8/1934 (H1N1)	A	EF467817.1
Cal7	A/California/7/2009 (H1N1)	A	Unpublished
Udorn72	A/Udorn/72 (H3N2)	A	CY009640.1
O175A^*a*^	A/green-winged teal/Ohio/175/1986 (H2N1)	A	CY018881.1
O173A	A/mallard/Ohio/173/1990 (H11N9)	A	CY021665.1
O340A	A/green-winged teal/Ohio/340/1987 (H11N9)	A	CY021873.1
M1124A	A/mallard/Maryland/1124/2005 (H11N9)	A	CY021473.1
O265B^*a*^	A/Mallard/Ohio/265/1987 (H1N9)	B	CY017279.1
O430B	A/green-winged teal/Ohio/430/1987 (H1N1)	B	CY011044.1
O264B	A/mallard/Ohio/264/1986 (H3N8)	B	CY016399.1
O339B	A/pintail/Ohio/339/1987 (H3N8)	B	CY019201.1
O668B	A/mallard/Ohio/668/2002 (H4N6)	B	CY020793.1
O671B	A/mallard/Ohio/671/2002 (H4N6)	B	CY020801.1
O35B	A/northern shoveler/Ohio/35/1986 (H3N8)	B	CY020937.1
O246B	A/bufflehead/Ohio/246/1986 (H11N2)	B	CY017079.1
Alb88B^*b*^	A/mallard/Alberta/88/1976 (H6N8)	B	M25373.1
NY6750A^*c*^	A/mallard/New York/6750/1978 (H2N2)	A	M25376.1
Sw418B^*d*^	A/mallard/Sweden/S90418/2005 (H6N8)	B	EU518722
NY107B^*e*^	A/New York/107/2003 (H7N8)	B	EU587374.2
J89B	A/equine/Jilin/1/1989 (H3N8)	B	M65020.1

a Used as representative consensus A- and B-allele genes for specific experiments.

b Residues 1 to 15 and 855 to 890 from ALB/221/1978 (H7N2) (GenBank accession no. CY005035.1). The G242A mutation was introduced to remove the BsmBI site.

c Residues 1 to 16 and 855 to 890 from A/mallard/New York/6750/1978 (H2N2) (GenBank accession no. M80945.1).

d Residues 1 to 26 and 882 to 890 from A/tufted duck/Mongolia/1409/2010 (H1N1) (GenBank accession no. KC871435.1). The A696C mutation was introduced to remove the BsmBI site.

e Residues 1 to 15 and 871 to 890 from A/guinea fowl/New York/20221-11/1995 (H2N2) (GenBank accession no. CY014833.1).

Polyclonal rabbit antisera to PR8 NS1 (antiserum V29), NEP (antiserum V13), nucleoprotein (NP; antiserum 2915), and matrix protein 1 (M1; antiserum 2917) have been described previously (41–44). Polyclonal rabbit antiserum against A/Mallard/Ohio/265/1987 (H1N9) (O265B) NS1 residues 211 to 226 (antiserum A2) was raised by inoculating rabbits with a keyhole limpet hemocyanin-peptide (CGPPLPPKQKRYMARRV) conjugate. A mouse monoclonal antibody to influenza A virus matrix 2 ion channel protein (M2) was purchased from Abcam (catalog number ab5416). Rat monoclonal antibody to α-tubulin was purchased from Serotec (clone YL1/2; catalog number MCA77G).

### Viral protein expression analysis.

MDCK cells in 24-well plates were infected at a multiplicity of infection (MOI) of 3 for 8 h and lysed in 200 μl SDS-PAGE loading buffer. Proteins were separated by SDS-PAGE and transferred to nitrocellulose membranes for Western blotting. Membranes were probed with primary antibody in phosphate-buffered saline–0.1% (vol/vol) Tween 20. The membranes were then probed with species-specific anti-IgG secondary antibodies conjugated to infrared-fluorescent dyes detectable using a LiCor Odyssey system. For radiolabeled immunoprecipitation, cells were infected at an MOI of 10, and at 4 h postinfection (p.i.), the medium was changed to Met- and Cys-free D-MEM containing 5% (vol/vol) dialyzed bovine serum. At 6 h p.i., cells were pulsed with 0.4 MBq ^35^S protein labeling mix (PerkinElmer) in Met- and Cys-free D-MEM. At 8 h p.i., the cells were lysed in a 0.1% SDS buffer (100 mM KCl, 50 mM Tris-HCl, pH 7.6, 5 mM MgCl_2_, 1 mM dithiothreitol, 1% Triton X-100, 1% sodium deoxycholate). Clarified lysates were incubated with rabbit polyclonal antiserum and protein A agarose (Roche). Immunoprecipitated proteins were eluted by boiling in SDS-PAGE sample buffer, separated by SDS-PAGE, and detected using autoradiography. For radiolabeling of whole-cell lysates, cells were infected at an MOI of 5 and radiolabeled as described above at 8 h p.i. After 1 h, the cells were lysed and the protein content was analyzed by SDS-PAGE and autoradiography.

### Type I interferon assays.

A549 cells were preconditioned with exogenous human beta interferon (Abcam) for 24 h prior to a multicycle infection (MOI, 0.01). At 48 h p.i., cell lysates were generated for Western blot analysis of viral NP and cellular interferon-induced GTP-binding protein Mx-1 (Mx-1), and virus in the supernatant was titrated by plaque assay. The 90% inhibitory concentration (IC_90_) values were calculated using GraphPad Prism software (v6).

To assay IFN production, 180 μl of an HEK-Blue cell suspension (at 2.8 × 10^5^ cells/ml) was added to 20 μl of a UV-treated (120,000 μJ/cm^2^ for 10 min; CL-1000 UV cross-linker; UVP) sample in 96-well plates and incubated for 24 h at 37°C. Twenty microliters of the HEK-Blue cell supernatant was transferred to 180 μl of Quanti-Blue substrate (InvivoGen) in a separate 96-well plate and incubated for 1 h at 37°C. The absorbance at 630 nm was recorded and used as a readout for active type I IFN levels in the initial samples. A standard curve was generated in parallel using known quantities of human IFN-β.

### Human and mouse cytokine protein arrays.

Human blood monocyte-derived macrophages (MDMs) were infected at an MOI of 1 for 24 h. Cytokine profiling of clarified supernatants was performed using a human cytokine array kit (human cytokine array kit, panel A; R&D Systems) and the near-infrared fluorescence detection protocol as instructed by manufacturer. Lung homogenates from BALB/c mice culled at day 4 p.i. were pooled and clarified, and cytokine profiling was performed using a mouse cytokine array kit (mouse cytokine array kit, panel A; R&D Systems) as described above.

### Quantitative proteomics.

Human lung A549 cells (1 × 10^7^) in 100-mm by 20-mm dishes were infected at an MOI of 5 for either 8 h, 16 h, or 24 h. Cells were lysed in a 6 M guanidine lysis buffer, and lysates were sonicated using a water bath sonicator. Lysates were clarified by centrifugation at 21,000 × *g* for 10 min and were frozen in liquid nitrogen. Lysates were then processed for mass spectrometry as previously described (45). Briefly, protein was digested with LysC and then trypsin. Peptides were labeled with tandem mass tag (TMT) reagents, and 12 fractions were generated by high-pH reversed-phase high-performance liquid chromatography. Mass spectrometry was performed using an Orbitrap Fusion mass spectrometer, and TMT reporter ions were quantified from the MS3 scan (46). Peptides were identified and quantified using a Sequest-based in-house software pipeline. The human UniProt database was searched, and peptide spectral matches were filtered to a 1% false discovery rate (FDR) using linear discriminant analysis in conjunction with the target-decoy method (47). The resulting data set was further collapsed to a final protein-level FDR of 1%. Protein assembly was guided by principles of parsimony. Proteins were quantified by summing TMT reporter ion counts across all matching peptide matches after filtering on the basis of isolation specificity. Reverse and contaminant proteins were removed, and protein quantitation values were exported for normalization and further analysis in Microsoft Excel software. Hierarchical centroid clustering based on an uncentered Pearson correlation was performed using Cluster software (v3.0; Stanford University) and visualized using Java TreeView software (http://jtreeview.sourceforge.net).

### Competition assays.

MDCK cells were infected with equal numbers of PFU of two viruses, and the progeny viruses were subjected to plaque assay under a 0.6% agarose overlay for 48 h. Individual plaque picks were amplified in MDCK cells, and 24 h later, the viral RNA in the supernatant was extracted using the TRIzol reagent (Life Technologies), while the cells were lysed in SDS-PAGE sample buffer. To identify the source of segment 8, cDNA was synthesized using avian myeloblastosis virus reverse transcriptase (Promega) and a primer complementary to the 3′ end of the PR8 NS segment vRNA (AGCAAAAGCAGGGTGAC) that also efficiently primes reverse transcription from segment 8 vRNA of all avian virus strains used in this study. RT-PCR was performed using *Taq* DNA polymerase (Invitrogen) and primers specific for the NS1 open reading frame of A/green-winged teal/Ohio/175/1986 (H2N1) (O175A) (forward primer, GCAACCGGTACCATGGATTCCAACACTGTGTC; reverse primer, GGTTGCACCGGTGTAACTTCTGACTCAATTGTTC) and O265B (forward primer, GCAACCGGTACCATGGACTCCAACACGATAACC; reverse primer, GGTTGCACCGGTGTAACTTCTGACTCAACTCTTC). Alternatively, the NS1 content of cell lysates was analyzed by SDS-PAGE and Western blotting, using the differential reactivity of rabbit polyclonal antisera V29 (which detects PR8 and O175A NS1) and A2 (which detects O175A and O265B NS1) to score plaque picks.

### Mouse infections.

BALB/c mice were purchased from Harlan UK Ltd. (Oxon, UK). All work was carried out under a UK Home Office license according to the Animals (Scientific Procedures) Act 1986. Five- to 6-week-old female mice were anesthetized using isoflurane (Merial Animal Health Ltd.) and infected intranasally with 500 PFU of virus in 40 μl serum-free D-MEM. The mice were weighed daily and assessed for visual signs of clinical disease, including inactivity, ruffled fur, and labored breathing. At days 2, 4, and 6 p.i., mice were euthanized by CO_2_ asphyxiation. The tip of the left lung was harvested into preservative (RNAlater; Life Technologies) prior to RNA extraction, while the remainder of the left lung was removed, homogenized in serum-free D-MEM, and clarified by centrifugation. The titers of infectious virus in the left lung were determined by plaque assay on MDCK cells. The lobes of the right lung were fixed in 10% (vol/vol) neutral buffered formalin, before being processed and then embedded in paraffin. Five-micrometer-thick sections were cut and stained with hematoxylin and eosin before being assessed (in a blind manner) by a pathologist (P.M.B.), and lesions were scored. The scoring system was as follows: 0, no lesions; 1, mild, focal inflammation and rare degeneration and necrosis; 2, moderate, multifocal inflammation with frequent necrotic cells; and 3, marked, multifocal inflammation with common necrosis and occasional fibrin accumulation. For immunofluorescence imaging, lung tissue was deparaffinized and stained for intracellular viral NP using rabbit polyclonal antiserum (antiserum 2915). The lung tissue was subsequently stained with an anti-rabbit IgG antibody conjugated with a fluorescent dye (Alexa Fluor 594; Life Technologies), prior to immunofluorescent imaging using a confocal microscope (model LSM 710; Zeiss).

### Mouse RT-qPCR array.

Custom TaqMan array plates (Applied Biosystems) were designed to analyze 3 RNA samples per plate, with 32 unique assays being performed per sample. Individual RT-quantitative PCR (qPCR) assays for cellular transcripts (see Table S1 in the supplemental material) were purchased from Applied Biosystems and were used according to the manufacturer's recommendations. Total RNA was extracted from the tip of the left lung of 3 mice per cohort at day 4 p.i. using an RNA extraction kit (QIAshredder; Qiagen) as instructed by the manufacturer. cDNA was synthesized from 25 ng total RNA with random hexamer primers using a high-capacity RNA-to-DNA reverse transcription kit (Applied Biosystems) according to the manufacturer's instructions. cDNA was loaded with TaqMan Universal Mastermix (Life Technologies) and run according to the manufacturer's instructions in a 7500 qPCR thermocycler (Applied Biosystems). Cycle threshold (*C_T_*) values were obtained using 7500 software (v2.0.6; Applied Biosystems) according to the manufacturer's instructions, and data were exported to Microsoft Excel software for further analysis. Raw *C_T_* values were normalized to GAPDH (glyceraldehyde-3-phosphate dehydrogenase) *C_T_* values (d*C_T_*) and plotted as 20 − d*C_T_*.

### Bioinformatics.

All segment 8 sequences were downloaded from GenBank via the Influenza Virus Resource (48) on 17 October 2014 (31,315 sequences). These data contained sequences isolated from avian (43%), swine (10%), human (44%), and other (3%) hosts. Due to the large number of sequences, the sequences were aligned by nucleotides in groups using the ClustalW tool in BioEdit software, manually adjusted for the NS1 coding frame with the remaining nucleotides in the NEP coding frame, and then aligned by amino acid sequence using the MUSCLE program in MEGA software (v6.0). The major lineages of influenza A virus segment 8 (avian virus A allele, human seasonal virus, classical swine virus, H1N1 pdm2009 virus, avian virus B allele) were identified in neighbor-joining trees; we split the data into 10 random subsamples of approximately 3,100 sequences each to make 10 trees, all the sequences belonging to the avian virus lineages were extracted from the random subsamples and combined, and the alignments were rechecked. Sequences with identical isolate names, mixed subtypes, unknown location, or year were excluded to make the avian virus A-allele (10,914 sequences in total, of which 9,617 were avian virus sequences) and avian virus B-allele (2,725 sequences in total, of which 2,717 were avian virus sequences) data sets (see Table S2 in the supplemental material for the distribution of sequences by all major lineages, hosts, geographic locations, and subtypes).

In order to determine the number of independent introductions from birds to mammals in the avian virus A-allele and avian virus B-allele data sets, we created phylogenetic trees using the neighbor-joining method in MEGA software (v6.0) (with the Tamura-Nei model, gamma-distributed rates among sites and heterogeneous patterns among lineages) in the first instance. For the avian virus B-allele data set, there were only 8 non-avian virus sequences in total, and by inspection, 2 pairs of them were the same or closely related in the tree, leaving a total of 6 introductions (Table 2).

**TABLE 2:** Independent introductions of avian virus B-allele NS segments into mammalian hosts

Strain	Host	Transmission	Notes (reference)	GenBank accession no. (NS segment)	Avian source
A/equine/Jilin/1/1989 (H3N8)	Equine	Epidemic	Major equine influenza epidemic (25)	M65020	Domestic
A/muskrat/Buryatiya/1944/2000 (H4N6)	Muskrat	Single isolate	Closely related to an H4N6 avian influenza virus circulating in Russia at a similar time point (70).	GU052363.1	Wild
A/swine/KU/16/2001 (H7N2)	Swine	Likely single isolate	Isolated from 1 pig (of 532 tested) from a slaughterhouse; apparent reassortment between avian H7N2 and H5N3 viruses (71).	CY067690	Domestic
A/swine/Saskatchewan/18789/02 (H1N1)	Swine	Likely transmissible in pigs	Isolated from a 1,200-sow pig farm where influenza-like symptoms affected pigs of all ages; fully avian virus (72)	AY619957	Wild
A/New York/107/2003 (H7N2)	Human	Single isolate	Apparent individual infection of a 48-year-old immunocompromised man from the Caribbean with fever and flu-like symptoms; no apparent transmission to family (73)	EU587374.2	Domestic
A/swine/Korea/C13/2008 (H5N2)	Swine	Apparent transmission	Serological evidence of transmission within pigs (maybe asymptomatic?) (74)	FJ461601.1	Wild

The avian virus A-allele data set was too large to make one tree for manual inspection; therefore, we subsampled the avian virus sequences (but not the non-avian virus sequences) to a maximum of 3 per year, subtype, host species type, and geographic region in order to make a tree of 4,886 sequences in total. In this tree, we manually identified sublineages where there were major introductions of an avian virus-derived segment 8 into mammals (old H7N7 equine virus, H7N7-H3N8 equine-canine viruses, Eurasian swine virus, H9N2 human and swine viruses, H7N9 viruses from human cases, H10N8 viruses from human cases, H3N2 canine virus, HPAI H5N1 virus) and then marked the other non-avian virus sequences in FigTree software (see Fig. S1 in the supplemental material).

For all of the 6 avian virus B-allele introductions, one representative sequence from each highlighted avian virus A-allele sublineage, and all the other avian virus A-allele non-avian virus sequences, a BLAST search for the 100 closest other sequences (or 500 other sequences in a minority of cases) was performed against the sequences in GenBank (using custom R scripts). The returned sequences for each BLAST search were analyzed, and the host, subtype, year, and geographic region properties for those which were isolated between 3 and 0 years before the query sequence was isolated (or 3 years before and after it was isolated if there were not sufficient returns) were extracted and summarized to estimate the source (domestic or wild birds) of the mammalian introductions. We also used an alternative means to estimate the source of the mammalian introductions: a neighbor-joining tree from the returned sequences was constructed (per query sequence), and a simple discrete trait model for host species (states consisting of domestic-galliformes, domestic-anseriformes, wild-anseriformes, wild-other, and nonavian), assuming equal rates, was fitted using the R package APE (49) to enable the inference of the avian host species of the ancestor of the mammalian virus sequence (or sequences). Finally, all the small trees generated from the BLAST searches were manually inspected, and searches of the literature were performed to obtain information about the query isolate. Results for nonindependently introduced isolates were removed, and a final list of independent mammalian introductions from avian sources was compiled by manual curation (Tables 2 and 3; see also Table S3 in the supplemental material).

**TABLE 3:** Independent introductions of avian virus A-allele NS segments into mammalian hosts

Prototypic strain	Host	Transmission	Notes	GenBank accession no. (NS segment)	Avian source
A/Brevig Mission/1/1918 (H1N1)	Human	Pandemic	Persists through classical swine, human H2N2, H3N2, and pdm2009 virus lineages	AF333238.1	Undetermined
A/equine/Prague/2/1956 (H7N7)	Equine	Transmissible	Likely to be extinct	CY087820	Undetermined
A/equine/Miami/1/1963 (H3N8)	Equine	Transmissible	Major circulating equine influenza A virus; also endemic in U.S. domestic dogs (canine H3N8 virus)	CY028840	Wild
A/swine/China/8/1978 (H3N2)	Swine	Circulating in pigs	Kawaoka et al. suggest that this belongs in an Eurasian avian virus lineage that is circulating in European pigs (75)	M80968	Domestic
A/swine/Belgium/WVL1/1979 (H1N1)	Swine	Transmissible	Representative isolate of Eurasian swine flu virus	CY037902	Domestic
A/seal/Massachusetts/1/1980 (H7N7)	Seal	Epidemic in seals	Epidemic in seals with approx 600 fatalities (76)	AB284067	Wild
A/seal/Massachusetts/133/1982 (H4N5)	Seal	Epidemic in seals	Responsible for a 1983 outbreak in which 60 seals were reported dead; 39 were positive for an H4N5 virus indistinguishable from the prototype virus (77)	M80947	Wild
A/mink/Sweden/3900/1984 (H10N4)	Mink	Outbreak in mink	One of the first identified cases of avian influenza virus able to transmit readily in mammalian host (78)	GQ176140	Domestic
A/whale/Maine/328B/1984 (H13N2)	Whale	Single isolate	Isolated from one sick pilot whale; no evidence of a transmission chain (79)	KJ372724	Wild
A/seal/Massachusetts/3911/1992 (H3N3)	Seal	Likely transmissible	Two closely related H3N3 viruses were detected in three seals following an increase in the number of stranded seals in Cape Cod in 1992 (80)	GU052287	Wild
A/swine/Hong Kong/644/1993 (H1N1)	Swine	Possible transmission.	Three independent H1N1 virus isolates from pigs with closely related NS segments; transmission information was not disclosed in the publication (81)	CY085013	Domestic
A/England/AV877/1996 (H7N7)	Human	Likely single isolate	Isolated from a 43-year-old duck farmer with mild one-sided conjunctivitis (82)	GU053113	Domestic
A/swine/Eire/89/1996 (H1N1)	Swine	Single isolate	No other closely related isolates of the same subtype, host, and year (83)	CY115892	Domestic
A/Hong Kong/482/97 (H5N1)	Human	Individuals only	Includes related H9N2 human and swine virus isolates in China 1997–2010	AF084285	Domestic
A/Swine/Ontario/01911-1/99 (H4N6)	Swine	Likely transmission	Responsible for an outbreak in pigs with pneumonia in Canada (84)	AF285889	Wild
A/swine/KU/2/2001 (H11N6)	Swine	Possible transmission	No published information available; four independent pig H11N6 virus isolates from the same year and location suggest ability to transmit within pigs	CY073456	Domestic
A/swine/Ontario/K01477/01 (H3N3)	Swine	Likely transmission	The same H3N3 virus was isolated from more than one pig in a sick group (exact numbers were not disclosed) (72)	AY619965	Wild
A/Caspian seal/Russia/1884/2002 (H4N6)	Seal	Possible transmission?	Two H4N6 virus isolates with very closely related NS segments were recovered from Caspian seals in Russia 10 yr apart; nothing was published for these strains	KJ847690	Wild
A/Netherlands/033/03 (H7N7)	Human	Poorly transmissible between humans	Mostly independent transmission events from birds to humans; H7N7 virus was epidemic in poultry in the Netherlands in 2003 (85); H7N7 IAV was detected in 85 subjects, with 3 people being from within a household (86, 87)	AY342423	Domestic
A/Beijing/01/2003 (H5N1**)**	Human	Not transmissible in humans?	Responsible for H5N1 bird flu; human-to-human transmission is rare or nonexistent	EF587281.1	Domestic
A/Canada/rv504/2004 (H7N3)	Human	Likely single isolates	Infection of two poultry workers on different farms during H7N3 poultry outbreak in British Columbia, Canada (88)	CY015010	Domestic
A/swine/Korea/S452/2004 (H9N2)	Swine	Likely single isolates	Two independent isolates of swine H9N2 viruses with very similar NS genes in 2004; the nature of infections was not specified in the literature (89)	AY790309	Domestic
A/canine/Guangdong/1/2006 (H3N2)	Canine	Transmissible	Avian H3N2 virus adapted to canine host and transmits well in population	GU433352	Wild
A/swine/Hubei/10/2008 (H10N5)	Swine	Transmissible in pigs	Wholly avian virus closely related to Eurasian swine IAV (90)	JX500447	Domestic
A/swine/Jilin/37/2008 (H3N2)	Swine	Likely single isolate	Authors sampled 279 sick pigs, and this was isolated from only 1 pig; has M and NS genes from H10 avian influenza virus (91)	GU215038	Wild
A/swine/Yangzhou/080/2009 (H6N6)	Swine	Likely transmissible in pigs	Detected from clinical samples coinfected with porcine circovirus type 2 in six pigs (92); closely related to A/swine/Guangdong/K6/2010 (H6N6); 3.6% of 475 sick pigs sampled were seropositive for H6 IAV (93)	JQ815882	Domestic
A/swine/HuBei/06/2009 (H4N1)	Swine	Single isolate	Apparent direct avian-to-pig infection without chain of transmission; the first H4 avian virus detected in pigs (94)	JX878679	Wild
A/swine/Guangdong/K4/2011 (H4N8)	Swine	Possibly transmissible in pigs	Isolated from a pig in a group displaying respiratory symptoms (H4N8 and H3N2) (95)	JX151011	Domestic
A/harbor seal/Massachusetts/1/2011 (H3N8)	Seal	Probable transmission	126 seals died from an outbreak caused by this virus (96)	JQ433883	Wild
A/Mexico/InDRE7218/2012 (H7N3)	Human	Likely single isolates	High-pathogenicity H7N3 virus isolated from Mexican poultry workers with conjunctivitis in 2012 (97); likely direct infection from infected poultry; the NS segment is similar to that of A/Canada/rv504/2004 (H7N3) (GenBank accession no. CY015010) (98)	CY125732	Domestic
A/Jiangxi/IPB13/2013 (H10N8)	Human	Likely single isolates	Human cases of H10N8 virus infection likely from live poultry markets in China (99); closely related to H7N9 precursor H9N2 strains	KJ406559	Domestic
A/Nanjing/1/2013 (H7N9)	Human	Poorly transmissible in humans	From 2013 and onwards, source of H7N9 virus zoonotic episodes in China	KC896778	Domestic

## RESULTS

### Avian virus A- and B-allele NS segment reassortants replicate efficiently in mammalian cells.

**FIG 1:**
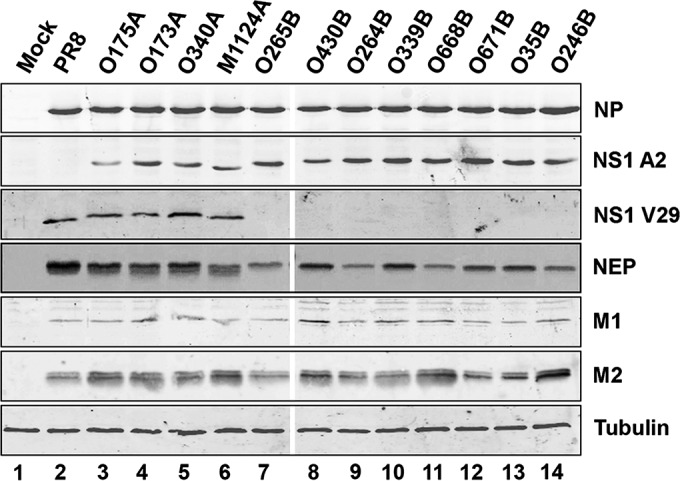
Protein synthesis by NS segment reassortant viruses in mammalian cell culture. MDCK cells were infected at an MOI of 3, and cell lysates were prepared at 8 h p.i. To detect viral NP, NS1, M1, and M2 polypeptides as well as cellular tubulin, lysates were subjected to SDS-PAGE and immunoblotted with the appropriate antisera. To detect NEP, cells were infected at an MOI of 10 and metabolically labeled with a ^35^S protein labeling mix between 6 h and 8 h p.i. The lysates were then immunoprecipitated with anti-NEP antiserum, and precipitates were analyzed by SDS-PAGE and autoradiography. Data are representative of those from more than one independent experiment.

As a first test of whether introduction of an avian virus-derived A- or B-allele segment 8 into a mammal-adapted virus introduced a fitness penalty in cultured mammalian cells, a panel of segment 8 reassortant viruses was generated on the background of PR8. The NS segments were taken from a variety of North American LPAI virus strains collected between 1986 and 2005 (Table 1) and were introduced into PR8 using a well-established reverse genetics system (34). The NS1 proteins of these viruses within their respective A- or B-allele group were at least 99% homologous and typically shared only 71% amino acid sequence homology between groups (see Fig. S2 in the supplemental material), consistent with previous reports (23, 24). All viruses were readily rescued. To assess viral protein expression during infection, MDCK cells were infected at an MOI of 3, and cell lysates were taken at 8 h p.i. and subjected to SDS-PAGE and Western blotting for viral NP, M1, M2, and NS1 proteins. All viruses produced comparable amounts of NP, M1, and, with some minor fluctuations, M2 (Fig. 1). NS1 from all viruses was also readily detected, with anti-PR8 NS1 serum V29 reacting with PR8 and the A-allele polypeptides and the B-allele serum A2 reacting with all avian virus NS1s. Avian virus NEPs could not be reliably detected by Western blotting with the available antisera (data not shown), but immunoprecipitation of radiolabeled cell lysates with the same serum provided unambiguous evidence of NEP expression by all viruses (Fig. 1). Thus, introduction of an LPAI virus strain NS segment of either the A- or B-allele lineage did not obviously attenuate viral protein expression in MDCK cells.

**FIG 2:**
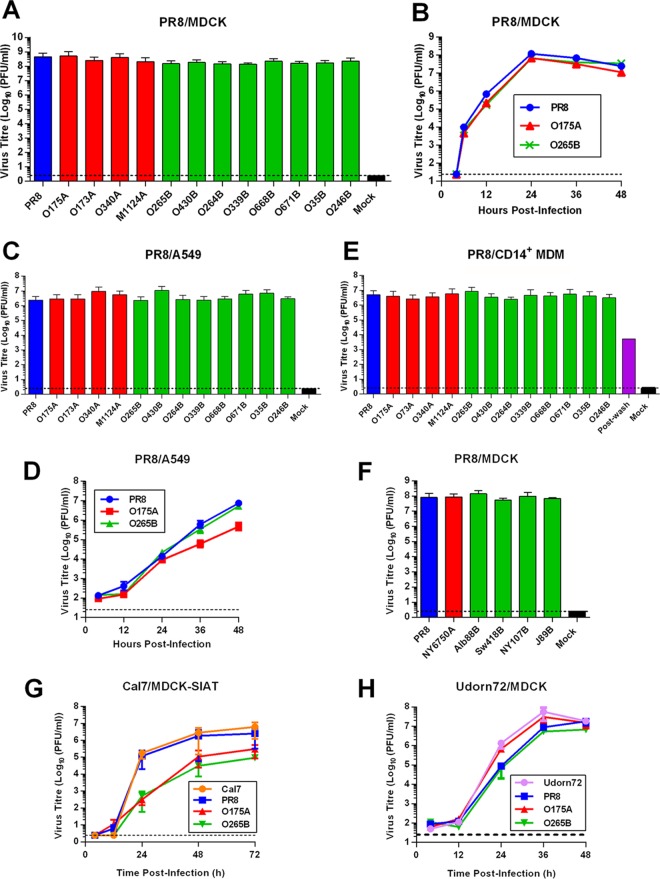
A- and B-allele reassortant viruses replicate efficiently in mammalian cell culture. (A, B) MDCK cells were infected at an MOI of 0.001 with PR8-based viruses, and the supernatants were titrated by plaque assay after 48 h (A) or at the plotted time points (B). Data in panel A are the mean ± SD (*n =* 5), while panel B presents the results of a single experiment. (C) A549 cells were infected with the indicated viruses at an MOI of 0.001, and endpoint titers were determined after 48 h. Data are the mean ± range (*n =* 2). (D) The replication kinetics of PR8 NS segment reassortants in A549 cells were determined as described in the legend to panel B. Data are the mean ± SD (*n =* 3). (E) Primary human CD14^+^ MDM cells were infected with PR8-based viruses at an MOI of 3, and the titers in the supernatant were determined after 24 h. Data are the mean ± range (*n =* 2). A duplicate sample of PR8-infected cells was taken immediately after the virus adsorption period (postwash), and titers were determined to confirm that the virus in samples collected at later times reflected true virus replication and not carryover of the virus inoculum. (F) Results of assays performed as described in the legends to panels A and B. Data represent the mean ± range (*n =* 2). (G) MDCK-SIAT cells were infected with Cal7-based viruses at an MOI of 0.01, and the supernatant was titrated at the plotted time points. Data are the mean ± range (*n =* 2). (H) MDCK cells were infected with Udorn72-based viruses as described in the legend to panel B. Data represent the mean ± range (*n =* 2). Dotted lines indicate the limit of detection.

To assess the replicative capabilities of the reassortant viruses in mammalian cells, MDCK cells were infected at an MOI of 0.001, and virus in the supernatant was titrated by plaque assay at 48 h p.i. All viruses replicated to greater than 10^8^ PFU/ml, and there were no statistically significant differences (assessed by an unpaired *t* test) between A- and B-allele reassortant virus titers (Fig. 2A). Additionally, the plaque phenotypes in MDCK cells were indistinguishable between the viruses (data not shown). We next investigated virus growth kinetics; MDCK cells were infected at an MOI of 0.001 with either wild-type (WT) PR8 or consensus A- and B-allele NS segment reassortants (O175A and O265B, respectively), and virus in the supernatant was titrated across a time course. All viruses replicated with similar growth kinetics, producing a peak viral titer of approximately 10^8^ PFU/ml at 24 h p.i. (Fig. 2B). To assess virus replication in continuous human cells, A549 cells were infected at an MOI of 0.001, and virus in the supernatant was assessed at 48 h p.i. All viruses replicated to similar endpoint titers of approximately 10^7^ PFU/ml (Fig. 2C). When the kinetics of replication of consensus reassortant viruses were examined in A549 cells, WT PR8 and O265B were essentially indistinguishable, while O175A replication slowed after 24 h to reach an endpoint titer that was approximately 1 order of magnitude lower than that of WT PR8 and O265B (Fig. 2D). Similarly, the whole panel of segment 8 reassortant viruses replicated to viral titers similar to the titer of WT PR8 following multicycle infection of human epithelial colorectal adenocarcinoma cells (data not shown). To assess replication in primary human cells, CD14^+^ monocyte-derived macrophages (MDMs) were infected at an MOI of 3, and virus in the supernatant was titrated at 24 h p.i. All viruses replicated to similar titers of greater than 10^6^ PFU/ml (Fig. 2E). Therefore, introduction of avian virus NS segments of both the A- and B-allele lineages does not necessarily attenuate PR8 virus replication in primary or continuous human cell lines.

Since we had failed to observe any significant attenuation of virus replication using the North American NS segment reassortant LPAI viruses, we generated further reassortant viruses using NS genes that spanned a wider date and geographical range and that either had been studied previously (18, 27) or were deemed to be of particular interest, such as the A/equine/Jilin/1/1989 (H3N8) (J89B) NS segment or the human isolate A/New York/107/2003 (H7N8) (NY107B) (Table 1). Reassortant viruses were generated on the PR8 background as before, and multicycle replication in MDCK cells was assessed. Again, all viruses replicated to titers approaching 10^8^ PFU/ml, with no obvious difference between viruses with the A and B alleles being detected (Fig. 2F).

To test if these results were specific to the laboratory-adapted PR8 backbone, we generated NS segment reassortant viruses on the backgrounds of the human pandemic H1N1 strain Cal7 or H3N2 strain Udorn72 and assessed multicycle growth in mammalian cells. MDCK-SIAT cells were infected with the Cal7 reassortant viruses at an MOI of 0.01, and infectious progeny virus was titrated at the time points indicated below by plaque assay. The WT Cal7 and the Cal7-PR8 reassortant viruses replicated with similar kinetics, with peak titers being 10^6^ PFU/ml at 72 h p.i. (Fig. 2G). In comparison, the segment 8 reassortant avian viruses displayed delayed growth kinetics and gave ∼10-fold lower peak titers at 72 h p.i. than WT Cal7. However, the replication kinetics between the Cal7-based A- and B-allele reassortant viruses were indistinguishable, providing no evidence for a B-allele-specific effect. All the Udorn72 viruses replicated in MDCK cells with similar growth kinetics, barring minor differences at 24 h p.i., giving peak titers of approximately 10^7^ PFU/ml (Fig. 2H). Overall, therefore, introduction of either avian virus A- or B-allele NS segments into human H1N1 and H3N2 strains does not generally attenuate replication in mammalian cells *in vitro*. This suggests that the NS1 proteins and NEPs of avian viruses of both the A- and B-allele lineages are able to carry out essential functions efficiently in mammalian cells during infection.

### A B-allele NS1 protein is efficient at controlling the mammalian innate immune response *in vitro*.

**FIG 3:**
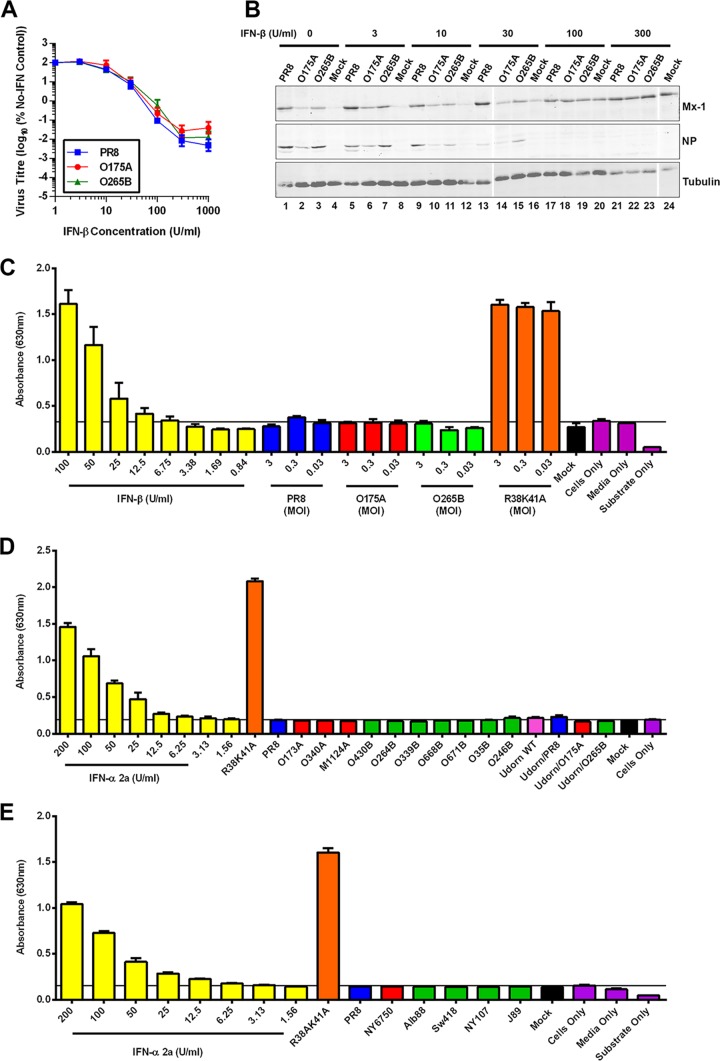
A B-allele reassortant is not deficient in controlling the host IFN response in mammalian cell culture. (A, B) Ability of viruses to replicate despite established antiviral conditions. A549 cells were pretreated with various concentrations of human recombinant IFN-β for 24 h prior to infection with the indicated PR8 viruses at an MOI of 0.01. (A) Virus in the supernatant was titrated by plaque assay at 24 to 48 h p.i. Data are the mean titers from 24-h and 48-h multicycle infections. (B) Cell lysates were prepared at 48 h p.i., subjected to SDS-PAGE, and immunoblotted for cellular IFN-inducible Mx-1, viral NP, and tubulin. (C to E) Induction of host cell type I IFN response during infection with reassortant viruses. (C) Human lung A549 cells were infected for 24 h at various multiplicities, and active type I IFN in the supernatant was quantified using the HEK-Blue reporter cell line. (D, E) Results of assay performed as described in the legend to panel C but at an MOI of 3.

Since it has been suggested that NS1 proteins belonging to the B-allele lineage cannot efficiently suppress a mammalian host innate immune response (26–29, 50), we investigated the ability of the consensus PR8 segment 8 reassortant viruses to circumvent and control the host cell type I IFN response. First, human A549 cells were preconditioned for 24 h with various concentrations of human IFN-β prior to infection at an MOI of 0.01. The endpoint titers of these multicycle replication reactions were then determined by plaque assay, while cell lysates were subjected to SDS-PAGE and Western blotting for Mx-1, viral NP, and tubulin. All viruses displayed a similar dose-response to exogenous IFN-β treatment, being largely insensitive to treatment with less than 10 U/ml but increasingly inhibited by higher doses (Fig. 3A). The IC_90_ values were very similar between PR8, O175A, and O265B (28.5 U/ml, 29.6 U/ml, and 33.5 U/ml, respectively). Increased concentrations of IFN-β pretreatment correlated with increased levels of the interferon-sensitive Mx-1 protein and decreased levels of viral NP (Fig. 3B). Therefore, both A- and B-allele NS segment reassortant viruses are as effective as a mammal-adapted virus at replicating in the presence of prior induction of the host innate immune response in mammalian cells. Next, we investigated the levels of type I IFN secreted from A549 cells in response to virus infection. A549 cells were infected for 24 h at an MOI of 3, 0.3, or 0.03 with WT and reassortant PR8 viruses or, as a positive control, with a PR8 NS1 mutant (R38K41A) known to be defective in its control of type I IFN expression (51, 52). The supernatants were then treated with UV light to inactivate virus, and the amount of type I IFN on HEK-Blue cells in comparison to the amount on a standard curve generated with known amounts of IFN-β was estimated by bioassay (53). Cells infected with the R38K41A virus at high or low multiplicities produced approximately 100 U/ml type I IFN (Fig. 3C). In contrast, WT PR8, O175A, and O265B infection (at any MOI tested) failed to induce levels of type I interferon significantly above the background level of the assay (approximately 6.75 U/ml IFN-β). We then assessed the full panel of PR8- and Udorn72-based reassortant viruses at an MOI of 3, and in no case was type I IFN detected in the supernatant of infected A549 cells at levels above the background levels (Fig. 3D and E). Thus, LPAI virus A- and B-allele NS1 proteins are able to effectively suppress type I IFN expression during infection to a level below that estimated to be inhibitory to virus replication, on the basis of dose-response curves of human IFN-β (Fig. 3A). Additionally, we tested various A- and B-allele NS1 proteins for their ability to block the activation of the IFN-β promoter following stimulation with poly(I·C) and found that all avian virus NS1s tested were sufficient in blocking this activation (data not shown). These data suggest that B-allele NS1 proteins are in fact able to successfully suppress the overall mammalian interferon response *in vitro*, contrary to previous reports (26–29).

**FIG 4:**
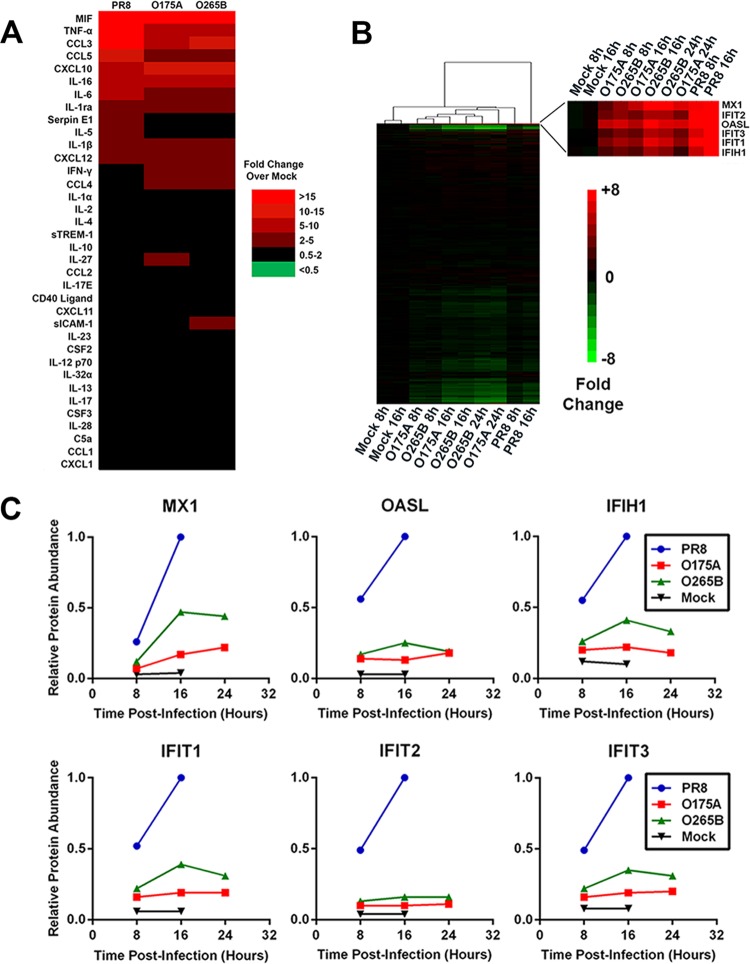
Induction of host innate immune response during infection with reassortant viruses. (A) Human CD14^+^ MDMs were infected at an MOI of 1 for 24 h, and the levels of various cytokines and chemokines in the supernatant were determined using an immunospot blot array. Values represent those from a heat map of the mean fold change in the level of expression with respect to the level in a mock-infected sample (Mock). sTREM-1, serum soluble triggering receptor expressed on myeloid cells-1; sICAM-1, soluble intercellular adhesion molecule-1; CSF2 and CSF3, colony-stimulating factors 2 and 3, respectively. (B, C) A549 cells were infected at an MOI of 5, cell lysates were generated at the indicated times, and the polypeptide composition was determined by TMT-based quantitative mass spectrometry. (B) Values represent those from a heat map of the mean fold change with respect to the value for mock-infected samples. The zoomed portion shows a subcluster of heavily upregulated antiviral proteins. (C) The quantitative temporal expression of specific antiviral restriction factors Mx-1, IFIT2, OASL, IFIT3, IFIT1, and IFIH1 is plotted.

We next looked at cytokine and chemokine secretion from infected primary human macrophages, since macrophages are important mediators of the host immune response to influenza virus infection (54, 55). Human CD14^+^ MDMs were infected at an MOI of 1, and the supernatant was harvested after 24 h. Successful infection was demonstrated by Western blotting for viral NP (data not shown). The levels of various secreted cytokines and chemokines following infection with PR8, O175A, or O265B were then quantified using an antibody capture array. Infection led to the upregulation of several cytokines and chemokines relative to the level of secretion in a mock-infected sample, with macrophage inhibitory factor (MIF), tumor necrosis factor alpha (TNF-α), chemokine (C-C) motif ligand 3 (CCL3), chemokine (C-C) motif ligand 5 (CCL5), C-X-C motif chemokine 10 (CXCL10), interleukin 16 (IL-16), interleukin 1 receptor antagonist (IL-1ra), interleukin 1β (IL-1β), and C-X-C motif chemokine 12 (CXCL12) all being upregulated at least 2-fold following infection with all three viruses (Fig. 4A). The O175A and O265B reassortant viruses produced similar secretome profiles, and in both of these samples the levels of proinflammatory mediators TNF-α, CCL3, and CCL5 were lower than those resulting from WT PR8 infection (Fig. 4A). Thus, there is no evidence to suggest that there is an elevated macrophage response to viruses with a B-allele NS segment in human macrophages *in vitro* in comparison to that to viruses with an A-allele counterpart.

To assess the global host cell response to infection with NS segment reassortant viruses, we measured changes in host cell proteins over a full time course of infection with each virus in A549 cells using a new technology, multiplexed tandem mass tag (TMT)-based mass spectrometry (45). This technology enables direct comparison of temporal infection with each virus within a single experiment. Cells were infected at an MOI of 5, and cell lysates were taken at 8 h, 16 h, and 24 h p.i. Immunofluorescent analysis of NP expression confirmed a mean infection rate of 95% across all virus-infected samples and time points (data not shown). A total of 6,862 unique proteins were quantified, and these were clustered according to the fold change in expression relative to that for the uninfected samples (Fig. 4B). The abundance of only a minority of cellular proteins changed significantly in response to infection, with the majority of these decreasing (see Table S4 in the supplemental material). However, the IFN-inducible factors Mx-1, interferon-induced protein with tetratricopeptide repeats 2 (IFIT2), 2′-5′-oligoadenylate synthetase-like protein (OASL), interferon-induced protein with tetratricopeptide repeats 3 (IFIT3), interferon-induced protein with tetratricopeptide repeats 1 (IFIT1), and interferon-induced protein with helicase C domain 1 (IFIH1) formed one example of a subcluster of highly upregulated host factors in the PR8-infected samples (Fig. 4C). The genes for these proteins were also induced in O175A- and O265B-infected cells, but generally to a lesser extent (Fig. 4C). Thus, WT PR8 was actually a greater inducer of host antiviral responses than either A- or B-allele NS segment reassortant avian viruses, suggesting that neither the A- nor B-allele NS1 proteins from avian viruses are deficient in controlling the mammalian innate immune response.

### Relative fitness of A- and B-allele segments.

**FIG 5:**
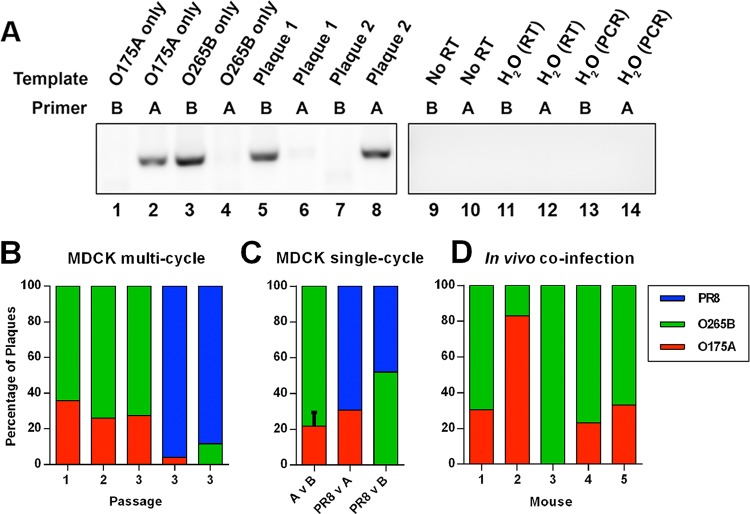
Relative fitness of PR8 reassortant viruses. (A) Establishment of a strain-specific RT-PCR assay for A- and B-allele viruses. Viral RNA was extracted following plaque purification of either O175A, O265B, or (as examples) 2 unidentified viruses obtained from coinfection with both and analyzed by RT-PCR with primers specific for the NS segment of either O175A or O265B. (B) MDCK cells were coinfected with the indicated mixtures of viruses at an MOI of 0.001 (each), and at 48 h p.i., the supernatant was analyzed by plaque assay. Twenty-five plaques were scored for the proportion of progeny, and a further portion of the original supernatant was passaged further at an MOI of 0.001. (C) MDCK cells were infected with each virus at an MOI of 3, and the proportion of each virus in the supernatant was assessed after 16 h (values are means + SEMs for avian virus with the A allele versus avian virus with the B allele [A v B]; *n =* 3). (D) Five BALB/c mice were coinfected with 250 PFU each of O175A and O265B. At day 6 p.i., the left lung of each mouse was harvested, and the proportion of each virus in the lung homogenate was determined by plaque purification of 12 plaques and RT-PCR.

To assess more subtle differences in replicative fitness between NS segment reassortant viruses, we performed mixed infections and then estimated the proportion of progeny virus that carried A- or B-allele NS segments. Initially, we tested whether, over the course of multiple rounds of replication in mammalian cells, the A-allele NS segment had a selective advantage in mammalian cells that would let it outcompete the B-allele NS segment. MDCK cells were infected with both O175A and O265B at an MOI of 0.001 each, and at 48 h p.i., clonal populations of progeny virus were isolated by plaque purification. Allele-specific RT-PCRs were then performed to identify the source of segment 8. Control reactions showed that a strong signal was obtained only with PCR mixtures containing homologous viral RNA and primers (Fig. 5A, lanes 1 to 4 and 9 to 14), such that individual plaques picked from coinfections could be unambiguously scored (see lanes 5 to 8 in Fig. 5A for examples). When 25 plaques from the coinfection were scored by this method, the B allele accounted for over 60% of the progeny (Fig. 5B). The output virus population was further passaged twice at an MOI of 0.001, and 25 plaques were assayed at each passage. O265B maintained an advantage over O175A through these multiple rounds of replication, forming 72% of plaques by passage 3 (Fig. 5B). When either avian virus segment 8 was similarly competed against the wild-type PR8 segment 8, PR8 heavily outcompeted both O175A (96% versus 4%) and O265B (88% versus 12%) by the end of the third passage (Fig. 5B). Thus, there was no evidence of even a small replicative fitness penalty for O265B compared to the fitness of O175A (if anything, the B allele appeared to win), but both avian virus segments were less fit than the PR8 gene.

Previously, we suggested that differences in the terminal packaging signal regions of segment 8 might restrict the incorporation of the B allele into an A-allele background (56). To test this, we performed high-multiplicity coinfections, such that the majority of infected cells would be expected to contain segment 8s of both backgrounds; the proportion of progeny virions containing an individual NS gene would therefore reflect the facility with which it was packaged into the PR8 virus background. MDCK cells were infected at an MOI of 3 per virus, and at 16 h p.i., progeny virus was collected and individual plaques were assayed as described above. When O265B was competed against O175A in three independent experiments, allele B-containing plaques outnumbered O175A plaques by approximately 3:1 (Fig. 5C). RT-qPCR analysis of intracellular segment 8 levels showed a 2.5:1 ratio in favor of the B-allele segment (data not shown), suggesting that the packaging efficiencies of O175A and O265B NS vRNA were similar. Similarly, when either of the avian virus segment 8s was competed against the PR8 segment in high-multiplicity coinfections, B-allele plaques were nearly equal in proportion with WT PR8 plaques (52% versus 48%), while WT PR8 modestly outcompeted O175A (69% versus 31%) (Fig. 5C). Therefore, contrary to our original hypothesis, there was no evidence for selective packaging of an A-allele segment into PR8 virions when both A and B segments were present in an infected cell.

We next looked at coinfection *in vivo*. Five BALB/c mice were intranasally infected with 250 PFU each of O175A and O265B. At day 6 p.i., the left lung of each mouse was harvested. Twelve plaques from each lung homogenate were assayed, and the proportion of O175A and O265B in the lung was estimated. In one sample, viruses bearing the O175A segment 8 predominated, but in all other samples, O265B provided the majority or even all isolates (Fig. 5D). On average, O265B outcompeted O175A by two to one (66% versus 34%) *in vivo*. Thus, there is no obvious selection advantage for an avian virus A-allele NS segment over a B-allele counterpart either *in vitro* or *in vivo*.

### A B-allele reassortant virus is more pathogenic than an avian virus A-allele counterpart *in vivo*.

**FIG 6:**
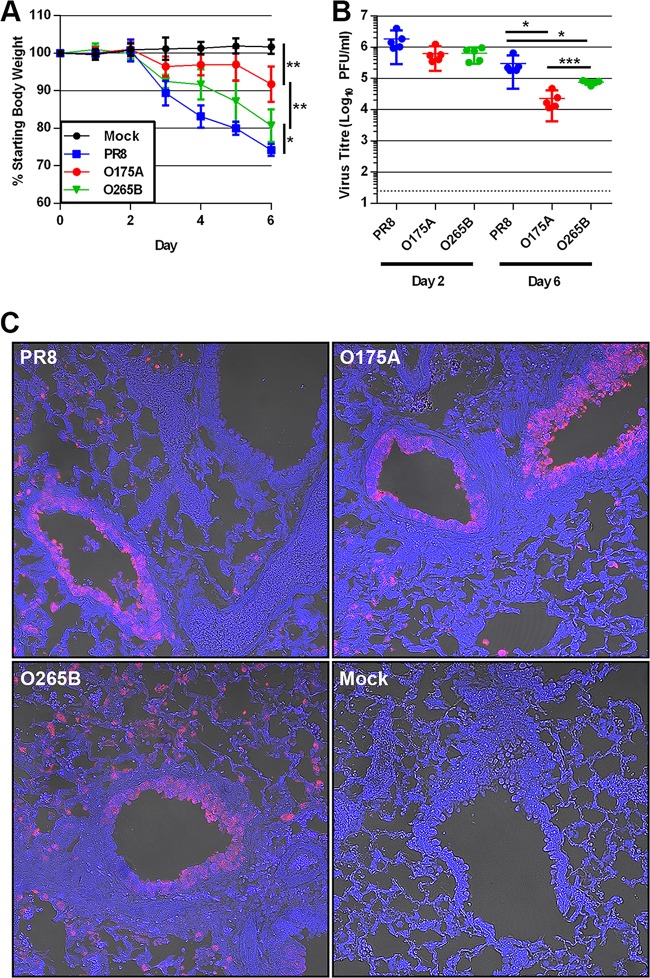
Pathogenicity of PR8 reassortant viruses in BALB/c mice. (A) Five BALB/c mice per group were nasally inoculated with 500 PFU of virus or mock infected with medium, and their weight was measured every 24 h. Data represent the mean ± SD. Asterisks indicate statistical significance at day 6 postinfection, when all animals were euthanized. (B) The left lung of each mouse was harvested at either day 2 or day 6 p.i., and virus in the homogenate was titrated by plaque assay. (C) Intracellular NP staining in infected mouse lung. Lung sections from mice at day 2 p.i. were stained for viral NP (red) and DNA (blue) and imaged using confocal and differential interference contrast (DIC) microscopy. *P* values were determined by unpaired *t* tests. *, *P* < 0.05; **, *P* < 0.01; ***, *P* < 0.005.

We assessed the ability of the consensus segment 8 reassortant viruses to replicate and cause disease *in vivo*. Groups of 5 BALB/c mice were challenged with 500 PFU of WT PR8, O175A, or O265B or mock infected with medium. Mock-infected mice maintained a healthy body weight over 6 days, whereas all infected mice lost weight from day 3 onwards (Fig. 6A). By this criterion, WT PR8 was the most virulent strain, causing the greatest weight loss, whereas both avian virus segment 8s apparently led to attenuated disease. Surprisingly, however, O265B induced more weight loss than O175A. The mean weight loss at day 4 p.i. was 16.9%, 3.1%, and 8.4% of the starting body weight for PR8-, O175A-, and O265B-infected mice, respectively (*n =* 10). By day 6 p.i., PR8, O175A, and O265B induced a mean weight loss of 25.8%, 8.2%, and 19.3%, respectively (*n =* 5). The weight loss in each group was significantly different from that in all other groups from day 3 p.i. onwards, with the exception of that in O175A-infected mice at day 5 p.i., in which the weight loss was not significantly different from that in mock-infected mice (unpaired *t* test) (Fig. 6A). The same outcome of PR8 causing the greatest weight loss and O175A causing the least was obtained in independent replicate experiments, including one with a lower inoculation dose of 100 PFU/animal, where infection with WT PR8 and the O265B reassortant gave mean nadir weights of 82.0% ± 1.0% (standard deviation [SD]) and 91.7% ± 2.1% (SD) of the initial body weight on day 7, respectively, before recovery, while O175A-infected animals gained weight throughout the experiment (data not shown). Thus, introduction of avian virus NS segments into PR8 attenuated disease *in vivo*, but this attenuation was the least with a B-allele segment.

To further assess replication characteristics *in vivo*, the lungs of infected mice were harvested at days 2 and 6 postinfection and homogenized, and virus titers were measured. At day 2 p.i., all three viruses had replicated to titers of about 1 × 10^6^ PFU/ml of homogenate, and while the average WT PR8 titer was approximately 3-fold greater than the titers of both O175A and O265B, the differences were not statistically significant (unpaired *t* test) (Fig. 6B). At day 6, the titers of all three viruses had dropped, but to significantly different extents, with WT PR8 giving the highest titers and O175A giving the lowest (unpaired *t* test). Thus, the source of segment 8 had relatively little effect on virus replication at early times postinfection but significantly affected virus clearance. As before, however, the avian virus-derived B allele was less attenuating than the avian virus-derived A allele. When lung sections obtained on days 2, 4, and 6 p.i. were examined by immunofluorescent staining for viral NP, in all cases, infected cells were found the most frequently in airway epithelia but also in interstitial tissue (Fig. 6C). In general, the number of positive cells correlated with the titers of infectious virus, with PR8-infected cells appearing to be more widespread than O265B- and O175A-infected cells (data not shown), but overall, the source of segment 8 did not noticeably affect virus tropism.

**FIG 7:**
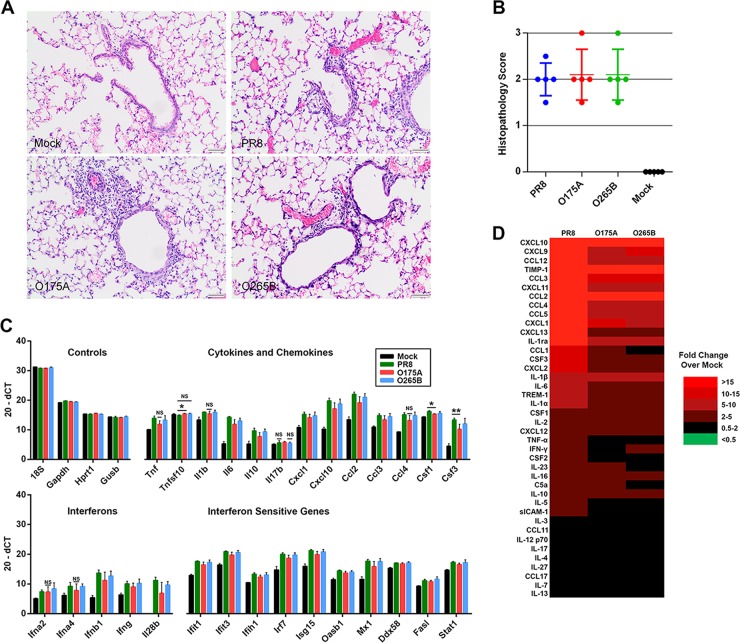
Host responses to A- and B-allele segment 8 reassortant viruses. (A) At day 6 p.i., the right lung lobes of inoculated mice were collected, fixed, processed, and stained with hematoxylin and eosin. Mock-infected mice showed no significant changes, whereas infected mice showed degeneration and necrosis of epithelial cells lining the airways with peribronchiolar and perivascular inflammation and interstitial inflammation sometimes accompanied by necrosis and fibrin accumulation. The inflammatory infiltrate was predominately lymphocytes and macrophages with variable numbers of neutrophils. Bars, 50 μm. (B) The severity of the pathology in the lung was assessed in a blind manner, and an overall pathology score out of 3 was assigned. (C) RNA was extracted from the lungs of infected mice at day 4 p.i., and the levels of various cytokines, chemokines, and antiviral gene expression were quantified using RT-qPCR. Data are plotted as the mean (20 − d*C_T_*) ± SD. The values for all infected samples were significantly different from the value for the mock-infected sample, unless the bar is labeled with NS, which indicates no significant difference by the unpaired *t* test. *, *P* < 0.05; **, *P* < 0.01. (D) The levels of various cytokines and chemokines in pooled lung homogenates from groups of 5 mice culled at day 4 p.i. were determined using an immunoblot spot array. Values are represented by those from a heat map of the fold change with respect to the level of expression for the mock-infected samples.

To assess and compare the severity of the pathology caused by each of the viruses, at day 6 p.i. the lungs of infected mice were fixed, processed, sectioned, stained with hematoxylin and eosin, and then examined to assess and score the histopathological changes present. All three viruses caused lesions consistent with IAV infection, characterized by mild to marked, subacute, multifocal, nonsuppurative, bronchointerstitial pneumonia with necrosis and fibrin accumulation. Degeneration and necrosis of the epithelial cells lining the airways, accompanied by peribronchiolar, peribronchial, and perivascular inflammation, were consistent features (Fig. 7A). The inflammatory infiltrate was predominantly lymphocytic and histiocytic with variable numbers of neutrophils. Scoring of the severity and extent of the changes revealed no difference in the pathology present in mice infected with PR8, O265B, or O175A (Fig. 7B).

To examine the antiviral response *in vivo* following infection with the reassortant viruses, we first quantified the expression of an array of antiviral genes (selected to represent cytokines and chemokines, interferons, and interferon-sensitive genes; see Table S1 in the supplemental material) in the lungs of infected mice at day 4 p.i. using RT-qPCR. The expression of most genes was significantly upregulated in infected mice in comparison to that in the mock-infected controls (Fig. 7C). In general, WT PR8 induced more antiviral gene expression than either NS segment reassortant virus, while O265B tended to induce antiviral gene expression more than O175A. However, the majority of the differences were not statistically significant, with the only exceptions being *Csf1* and *Csf3*, both of which were significantly upregulated by WT PR8 in comparison to their level of regulation in O175A-infected mice (*n =* 3; *P* < 0.05, unpaired *t* test). There were no instances of gene expression being significantly different between O175A- and O265B-infected lungs. To check for the possibility of biologically significant posttranscriptional regulation, we next created proteome profiles of infected mouse lung (using pooled lung homogenates from groups of 5 mice) at day 4 p.i. for various cytokines and chemokines using an antibody capture array. WT PR8 infection induced higher levels of proinflammatory cytokines, such as TNF-α, CXCL12, and interleukin 6 (IL-6), than infection with the segment 8 reassortant viruses, while the profiles in O175A- and O265B-infected mice were more similar to each other than to the profile in PR8-infected mice (Fig. 7D). Thus, *in vivo* as well as *in vitro*, WT PR8 was the strongest inducer of the host antiviral response, but in this background, an avian virus B-allele segment 8 caused greater disease than an A-allele counterpart and allowed prolonged virus persistence in the host.

### The B-allele NS gene of influenza A virus reassorts into mammal-adapted viruses at similar rates as the A-allele NS gene.

The behavior of our reassortant viruses failed to provide evidence that the B-allele NS segment is especially maladapted to a mammalian host. However, this hypothesis is founded on the presumption that B-allele segment 8s are rarely found in viruses isolated from mammals, a view dating back over 25 years, when there were far fewer IAV sequences available. We therefore decided to revisit the question of how often avian virus-derived segment 8 jumps into mammalian hosts, using the much larger number of virus sequences now available.

**FIG 8:**
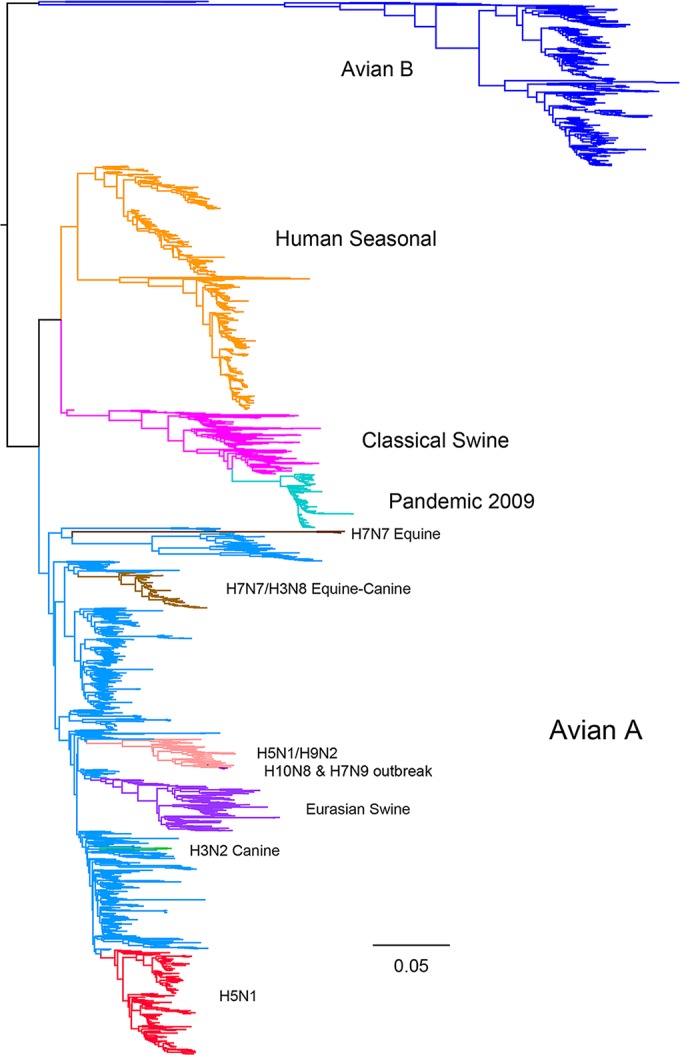
Major lineages of influenza A virus segment 8. A phylogenetic tree of a subsample of all influenza A virus segment 8 sequences is shown, with the major lineages being highlighted in color.

**FIG 9:**
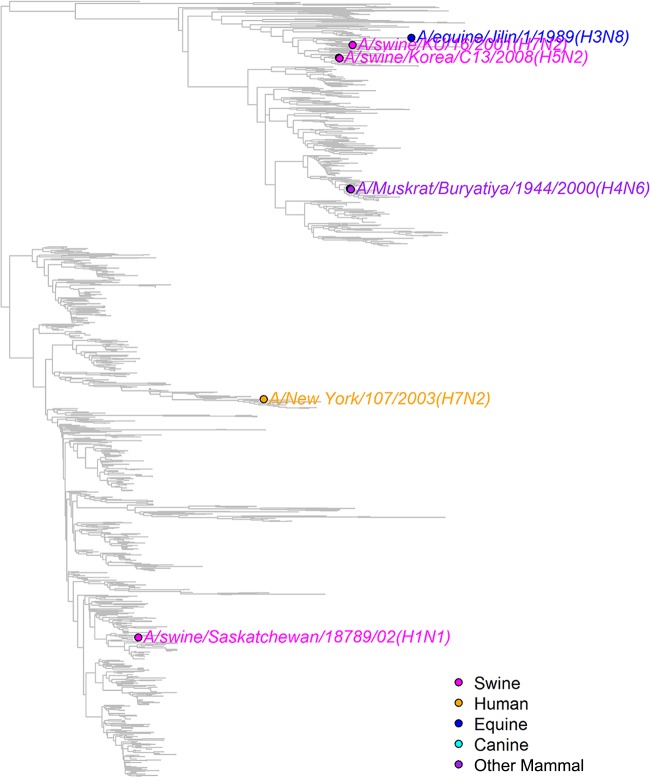
Mammalian virus B-allele segment 8 sequences. A phylogenetic tree of all avian virus B-allele lineage segment 8 sequences is shown, and the non-avian virus sequences are highlighted.

**FIG 10:**
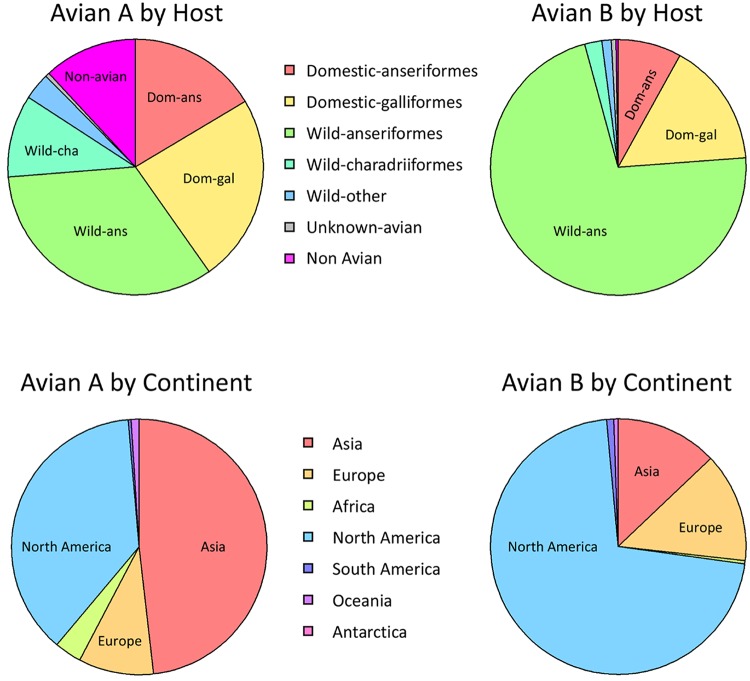
Distribution of avian virus segment 8 sequences. The host distribution and location of isolation are represented for all avian virus A- and B-allele segment 8 sequences.

All NS segment sequences in GenBank were downloaded and grouped into A or B alleles (see Materials and Methods and Table S2 in the supplemental material). These sequences were phylogenetically clustered to identify the major lineages of influenza A virus segment 8 (Fig. 8), including several obvious large clades of isolates from mammalian hosts that had likely acquired an NS segment from an ancestral avian IAV strain (e.g., human seasonal/classical swine/pdm2009 virus, Eurasian swine virus). To identify other bird-to-mammal transmission events, the trees were examined for phylogenetically incongruent examples of mammalian virus isolates within otherwise avian virus-derived branches (Fig. 8 and 9; see also Fig. S1 and Table S3 in the supplemental material). In some instances, these apparently represented single transmission events (when the limitations of sample bias are accepted), whereas others clearly reflected the successful introduction of an avian virus segment into a mammalian virus population. Since we wanted to estimate the numbers of independent introductions of an avian virus A- or B-allele NS segment into mammalian viruses, the latter class was still treated as one event. This approach identified 6 occasions in which a B-allele NS segment had demonstrably transferred to a mammalian host (Fig. 9; Table 2) and 32 occasions for A-allele NS segments (Table 3; see also Fig. S1 in the supplemental material). Thus, A-allele jumps from Aves to Mammalia hosts outnumber B-allele jumps by over 5 to 1. However, A- and B-allele segments may not be equally distributed among the source population, as the total number of avian virus A-allele sequences available in GenBank outnumbers the number of B-allele sequences available by approximately 3.5 to 1 (Table 4; see also Table S2 in the supplemental material). Using Fisher tests, we found that the number of mammalian introductions per avian virus isolate was not significantly different between the A and B alleles (A allele, 32 from 9,617; B allele, 6 from 2,717; *P* = 0.436). We further considered if there might be a difference in the detected number of introductions depending upon whether the avian source was wild or domestic birds, since the contact rate between the often sampled domestic mammals (e.g., humans and swine) and domestic birds could be very different from that for wild birds. Indeed, a larger proportion of the avian virus B-allele gene sequences than avian virus A-allele gene sequences were sampled from wild birds and from North America (Fig. 10). However, there was also no significant difference between A and B alleles considering likely domestic bird-to-mammal (*P* = 0.756) or wild bird-to-mammal (*P* = 0.578) introductions (Table 4). Thus, overall, the currently available surveillance/sequencing data do not support the hypothesis that the B-allele NS segment is specifically restricted to mammalian hosts but instead suggest that the introduction of any avian virus segment 8 into mammals is a relatively rare event.

**TABLE 4:** Expected number of avian B-allele introductions

Data	A allele			B allele			Fisher test^*a*^	
	No. of avian virus strains	No. of independent introductions	Introduction rate	No. of avian virus strains	No. of independent introductions	Introduction rate	*P* value	Expected no. of B-allele introductions^*b*^
All avian viruses	9,617	32	0.0033	2,717	6	0.0022	0.436	9.04 (4–15)
Transmissible viruses	9,617	19	0.0020	2,717	3	0.0011	0.446	5.37 (0–10)
Single isolates or viruses with poor transmission	9,617	13	0.0014	2,717	3	0.0011	1.000	3.67 (0–8)
Viruses from domestic birds only	4,391	18	0.0041	648	3	0.0046	0.756	2.66 (0–6)
Viruses from wild birds only	5,168	12	0.0023	2,053	3	0.0015	0.578	4.77 (1–9)

a Calculated using data from A alleles, assuming a binomial distribution.

b Data in parentheses represent the range of the number of episodes that would still be statistically nonsignificant (*P* ≥ 0.1).

## DISCUSSION

To date, there has been conflicting evidence regarding the fitness of IAV strains containing the B allele of NS segment 8 in mammalian hosts, from early evidence for restriction in mammals through to more recent studies that have begun to question this (18, 26–30, 57). Here, we found no major fitness penalty incurred *in vitro* or *in vivo* by H1N1 and H3N2 mammal-adapted influenza viruses forcibly reassorted with B-allele NS segments. We tested three mammal-adapted backbone viruses and a panel of A- and B-allele NS segments from a variety of isolates, predominantly LPAI virus strains from North America (Table 1); all viruses replicated to high peak titers in a variety of mammalian cell lines and achieved normal viral protein expression (Fig. 1 and 2). NEP is essential for virus replication (58), while deletion of NS1 severely attenuates virus replication in cells (59). Thus, despite their divergent avian virus origin, the B-allele NS1 proteins and NEPs are both able to effectively carry out essential support functions in mammalian cells *in vitro*. Indeed, in competition assays *in vitro* and *in vivo*, the B-allele reassortant consistently outperformed its avian virus A-allele counterpart (Fig. 5).

There is evidence, largely based on transfection-based reporter assays examining the function of individual cellular promoters, that B-allele NS1 proteins are less effective than their A-allele counterparts at suppressing the activation of specific innate immunity pathways in mammalian cells (26–29, 50). Consistent with these data, we also found significant differences in the ability of certain avian virus NS1s to antagonize RNA polymerase II-mediated gene expression from transfected plasmids with simian virus 40 or the interferon-sensitive response element but not the IFN-β promoters (data not shown). However, in the context of virus infection, both A-allele and B-allele H1N1 and H3N2 reassortant viruses controlled the secretion of active type I IFN from infected human lung cells to below an inhibitory level (Fig. 3C to E). These observations are complemented by those from a previous report showing that avian virus NS1 proteins from both the A- and B-allele lineages were able to antagonize type I IFN induction in human cells in infection-based reporter studies (60). Additionally, O175A and O265B displayed no obvious hypersensitivity to IFN-β pretreatment in human lung cells (Fig. 3A) and did not stimulate a large cytokine/chemokine response in primary human macrophages (Fig. 4A). In fact, both avian virus segment 8 reassortants induced less of an antiviral response than wild-type PR8 virus *in vitro* in human lung cells (Fig. 4B and C) and *in vivo* in mouse lungs (Fig. 7C and D). Thus, both A- and B-allele NS1 proteins from LPAI virus strains can effectively control the overall mammalian innate immune response and circumvent an induced antiviral state, even if, perhaps, the suite of mechanisms deployed shows some differences.

We also considered whether penetration of the B-allele segment into mammalian strains of virus might be restricted at the level of reassortment. IAV is known to package each segment specifically, with RNA packaging signals directing the process (56, 61). The packaging signals for segment 8 partially overlap variable regions between the alleles, and we previously hypothesized that this might hinder free reassortment of the avian virus B-allele segment into an otherwise mammal-adapted virus background (56, 62). However, the outcome of high-multiplicity coinfections with the A- and B-allele viruses, in which every cell would be expected to contain both segment 8s, which would then compete for incorporation into the A-allele PR8 background, did not support the hypothesis, with the B allele outperforming the avian virus A allele and drawing against the PR8 segment (Fig. 5C). This outcome is consistent with that in other work indicating a surprising amount of plasticity in the segment 8 packaging signal (63). Overall, the data therefore do not suggest that a B-allele NS vRNA segment is less likely to be introduced into an IAV strain circulating in mammalian hosts by genetic reassortment in nature.

Having failed to find any convincing evidence for a replicative penalty for the B-allele NS segment *in vitro* or *in vivo*, we began to question the validity of the long-held view that the B allele is restricted in mammals. There is evidence to suggest that the B-allele NS segment of IAV attenuates a human IAV strain *in vivo*. Treanor et al. made human H3N2 NS reassortant viruses and noted an attenuation in virus replication in the nasopharynx of squirrel monkeys and a reduced shedding duration of a B-allele reassortant virus in comparison to that for an A-allele counterpart (18). However, the peak titer of the B-allele reassortant virus in the trachea was not significantly reduced, suggesting that the virus could replicate effectively in this part of the respiratory tract. Here, we challenged BALB/c mice with PR8-based NS reassortant viruses and found that PR8, O175A, and O265B were all able to cause clinical disease and replicate *in vivo*. Wild-type PR8 caused the most severe disease in terms of weight loss (Fig. 6A), and this could be due to a greater induction of cytokines, chemokines, and interferon-sensitive genes in the lungs of infected mice (Fig. 7C and D) or due to prolonged viral persistence in the lung, as judged by the viral titers at day 6 p.i. (Fig. 6B). It is not surprising that wild-type PR8 caused the greatest disease, since this strain is already mouse adapted (64) (our PR8 clone has a 50% mouse lethal dose [MLD_50_] of ∼100 PFU), and it is known that the NS1 and NEP genes incur adaptive mutations following passaging in mice (17). Nevertheless, both avian virus NS reassortants caused overt disease, including indistinguishable levels of classical IAV-induced lung histopathology. O265B induced more severe weight loss than O175A; however, the cytokine, chemokine, and interferon-sensitive gene profiles in the infected mouse lung were not significantly different between the two. PR8, O175A, and O265B had similar peak viral titers at day 2 p.i., and the rate of clearance was O175A > O265B > PR8, which was inversely correlated with the severity of weight loss (Fig. 6B). Thus, in this mammalian infection model, a B-allele NS segment is compatible with high levels of virus replication and pathogenicity. Our study is not alone in finding that a B-allele segment is compatible with virulence in mice; a recent study found that insertion of a B-allele NS segment into a PR8 variant with a markedly higher MLD_50_ of ∼10^5^ PFU did not further attenuate the virus (65), while others found that insertion of a B-allele NS segment into a previously avirulent laboratory-adapted HPAI virus strain actually caused a dramatic increase in pathogenicity (30). Thus, overall, B-allele-containing viruses seem to be entirely capable of supporting substantial levels of virus replication and disease in laboratory animal models.

Twenty years ago, the other main strand of evidence for host restriction of B-allele NS genes was the paucity of mammal-derived isolates (23, 24, 66). However, the number of sequenced IAV isolates has since risen exponentially, and following the first report of a mammalian (equine) B-allele-containing virus (25), a number of other instances of B-allele-containing virus from swine and other species, including humans, have been noted (Table 2). It was therefore important to reappraise the question of whether the introduction of avian virus-derived NS segments into mammalian hosts is truly biased against one allele or another. We identified ∼12,000 unique avian virus-derived NS sequences in GenBank, of which over three-quarters were A-allele segments. A parallel analysis of mammal virus-derived NS sequences for phylogenetically incongruent sequences that represented introduction of an avian virus gene into a mammalian host, as well as consideration of well-established histories of IAV evolution (most notably, the 1918 avian virus NS segment, which founded the widespread classical swine, human H1N1, H2N2, H3N2, and pdm2009 virus lineages from a single event), identified 32 separate instances of an avian virus A allele and 6 of an avian virus B allele jumping into mammals. This gives rates of 32/9,617 and 6/2,717, respectively, or 0.0033 and 0.0022, respectively, for mammalian incursions per unique avian virus isolate (Table 4). These estimates are potentially affected by biases inherent to the data, most especially, sampling bias. The B allele might truly be less prevalent in the avian population, or it might preferentially infect bird species that are less frequently sampled (Fig. 10). For example, Adams et al. reported that a B-allele reassortant was less competent than an A-allele equivalent at replicating in chicken and turkey cells but was more fit in duck cells (67). However, surveillance data show that B-allele influenza viruses can infect poultry (Fig. 10) and have the ability to persist (68) and to be highly pathogenic (69). Conversely, the mammalian isolates are undoubtedly biased in the species that are sampled. We also accept that the boundary of an independent introduction can be defined in different ways. For instance, while forward transmission of the original avian virus-derived equine H3N8 virus to dogs clearly does not count as two independent introductions (going from bird to mammal to mammal), how does one define the repeated zoonotic infection of a widespread avian virus, such as H5N1, into humans? We chose to define this as two major introduction events, the original 1997 event and the subsequent reintroduction from domestic poultry in 2003, since the multiple human infections were monophyletic in each case. Nevertheless, taken on face value, our analysis of the currently available sequences does not support the hypothesis of the B allele being specifically restricted to avian hosts. Instead, it seems that the transfer of any avian virus segment 8 to mammals is a relatively rare event.

Successful cross-species transmission does not necessarily equate with establishment in the new host. Further inspection of the Aves-to-Mammalia transmission events suggests a divide into isolated events with little or no documented onward transmission in the mammalian host and those where the virus containing the avian virus segment 8 has persisted, at least for a period. The available information suggests that 3 of 6 B-allele viruses were transmitted between mammals, most notably, the 1989 H3N8 outbreak in horses (Table 2) (25). For the A-allele viruses, perhaps 19 out of 32 seem to have been transmissible, with half a dozen or more events leading to the long-term establishment of the virus in a new host. Statistically, these data do not support the hypothesis that the transmissibility of IAV with allele A or B NS segments is different (Table 4). However, the number of observed introductions is small, and there may be confounding effects from other segments. Further work would be necessary to test the possibility that transmission between mammalian hosts is attenuated due to the B-allele NS segment. Overall, we conclude that it is misleading to simply consider the B allele of the NS segment to be particularly restricted to avian host species, a factor to be borne in mind when assessing the risk of the zoonotic/epizootic potential of avian influenza strains (31).

## Supplementary Material

Supplemental material
