# Supplementary material for: Role of the B Allele of Influenza A Virus Segment 8 in Setting Mammalian Host Range and Pathogenicity
Source: J Virol. 2016 Sep 29;90(20):9263–84. doi: 10.1128/JVI.01205-16 (PMC5044859; doi:10.1128/JVI.01205-16)
Supplement: Supplemental material [file supp_90_20_9263__index.html]

Role of the B Allele of Influenza A Virus Segment 8 in Setting Mammalian Host Range and Pathogenicity — Supplemental material 

# Role of the B Allele of Influenza A Virus Segment 8 in Setting Mammalian Host Range and Pathogenicity

## Supplemental material

- Supplemental file 1 -

  Table S1 (Individual cellular transcript assays for mouse RT-qPCR array.)

  Table S2 (Distribution of sequences.)

  Table S3 (Details of mammalian introductions.)

  Fig S1 (Neighbor joining tree of a subsample of avian A virus sequences with sublineages highlighted; see also separate file below.)

  Fig S2 (NS1 protein alignment.)

  PDF, 3.0M
- Supplemental file 2 -

  Table S4 (Relative abundance over time of each protein quantified, during infection of A549 cells.)

  XLSX, 1.2M
- Supplemental file 3 -

  Fig. S1 (This is a separate file viewable in FigTree (http://tree.bio.ed.ac.uk/software/figtree/), which allows large phylogenetic trees to be viewed on screen at a magnification that permits virus strain names to be read.)

  XLS, 997K
