# Supplementary material for: Role of the B Allele of Influenza A Virus Segment 8 in Setting Mammalian Host Range and Pathogenicity
Source: J Virol. 2016 Sep 29;90(20):9263–84. doi: 10.1128/JVI.01205-16 (PMC5044859; doi:10.1128/JVI.01205-16)
Supplement: Supplemental material [file JVI.01205-16_zjv999182012so1.pdf]

Turnbull et al: Supporting information

File list (this pdf):

Table S1. Individual cellular transcript assays for mouse RT-qPCR array.

Table S2. Distribution of sequences.

Fig S1. Neighbour joining tree of a subsample of Avian A sequences with sub-lineages highlighted.

Table S3. Details of mammalian introductions.

Fig S2. NS1 protein alignment.

Uploaded separately:

Table S4. Relative abundance over time of each protein quantified, during infection of A549 cells.

Fig S1.xls. This is a separate file viewable in the programme figTree (downloadable in Mac and PC versions from http://tree.bio.ed.ac.uk/software/figtree/) which allows large phylogenetic trees to be viewed on screen at a magnification that permits virus strain names to be read.

Table S1. Individual cellular transcript assays for mouse RT-qPCR array. Individual RT-qPCR assays are available online (www.lifetechnologies.com).

Cellular Transcript	Taqman® Assay ID
18S	Hs99999901_s1
Gapdh	Mm99999915_g1
Hprt1	Mm00446968_m1
Gusb	Mm00446953_m1
Ifna2	Mm00833961_s1
Ifna4	Mm00833969_s1
Ifnb1	Mm00439546_s1
Igng	Mm00801778_m1
Il28b	Mm00663660_g1
Tnf	Mm00443259_g1
Tnfsf10	Mm00437174_m1
Il1b	Mm01336189_m1
Il6	Mm00446190_m1
Il10	Mm00439615_g1
Il17b	Mm00444686_m1
Cxcl1	Mm00433859_m1
Cxcl10	Mm00445235_m1
Ccl2	Mm00441242_m1
Ccl3	Mm00441259_g1
Ccl4	Mm00443111_m1
Csf1	Mm00432686_m1
Csf3	Mm00438335_g1
Ifit1	Mm00515153_m1
Ifit3	Mm01704846_s1
Ifih1	Mm00459183_m1
Irf7	Mm00516788_m1
Isg15	Mm01705338_s1
Oas1b	Mm00449297_m1
Mx1	Mm01217998_m1
Ddx58	Mm00554529_m1
Fasl	Mm00438864_m1
Stat1	Mm00439531_m1

Table S2 : Distribution of Sequences

NS sequences from GenBank with unique isolate names and Host, Subtype and Location information.
Excludes environmental samples and mixed subtype samples.

Lineage	AvianA	AvianB	ClassicalSwine	HumanSeasonal	Pandemic2009	All
Total	10914	2725	1872	7213	6098	28822

Host	AvianA	AvianB	ClassicalSwine	HumanSeasonal	Pandemic2009	All
Avian	9613	2717	22	2	5	12359
Swine	519	4	1807	72	561	2963
Human	325	1	38	7136	5518	13018
Equine	178	1	1	0	0	180
Canine	232	0	0	2	6	240
OtherMammal	47	2	4	1	8	62

Continent	AvianA	AvianB	ClassicalSwine	HumanSeasonal	Pandemic2009	All
Asia	5263	351	431	1700	1762	9507
Europe	1038	378	7	523	1241	3187
Africa	372	11	0	67	62	512
NorthAmerica	4092	1945	1434	3277	2408	13156
SouthAmerica	36	25	0	757	438	1256
Oceania	109	15	0	889	187	1200
Antarctica	4	0	0	0	0	4

H-subtype	AvianA	AvianB	ClassicalSwine	HumanSeasonal	Pandemic2009	All
H1	769	172	1176	1616	5924	9657
H2	321	87	3	98	0	509
H3	1294	648	692	5498	174	8306
H4	876	494	0	0	0	1370
H5	2981	337	0	1	0	3319
H6	1131	275	0	0	0	1406
H7	845	374	1	0	0	1220
H8	113	25	0	0	0	138
H9	1359	64	0	0	0	1423
H10	580	63	0	0	0	643
H11	387	141	0	0	0	528
H12	116	37	0	0	0	153
H13	92	1	0	0	0	93
H14	8	6	0	0	0	14
H15	8	1	0	0	0	9
H16	34	0	0	0	0	34

N-subtype	AvianA	AvianB	ClassicalSwine	HumanSeasonal	Pandemic2009	All
N1	3492	453	767	1575	5811	12098
N2	2763	681	1101	5638	287	10470
N3	751	135	3	0	0	889
N4	206	43	0	0	0	249
N5	228	93	0	0	0	321
N6	1111	467	0	0	0	1578
N7	677	66	0	0	0	743
N8	1113	652	1	0	0	1766
N9	573	135	0	0	0	708

Fig S1. Neighbour joining tree of a subsample of Avian A sequences with sub-lineages highlighted.

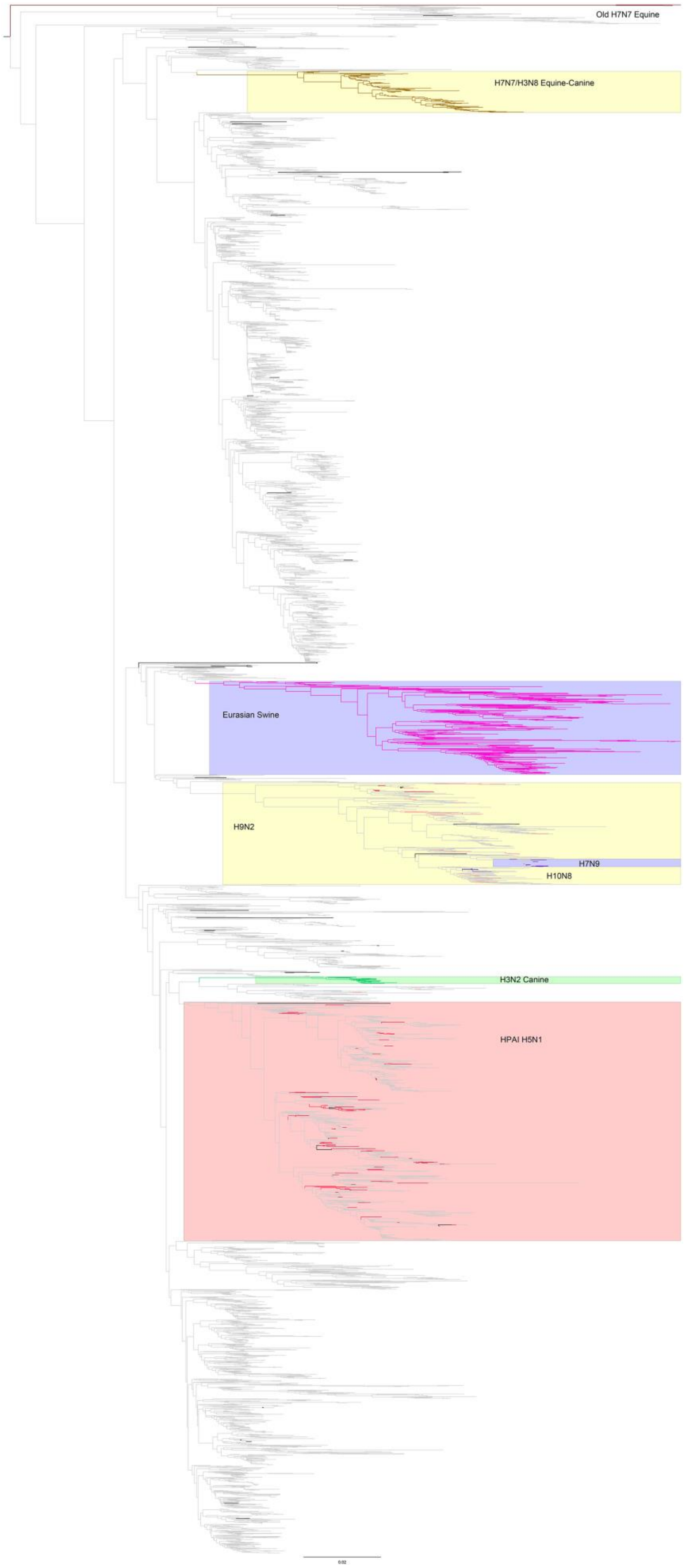

Table S3 : Details of mammalian introductions - source information obtained by analysing sequences returned by BLAST

Domestic Source							Ancestral State Estimation		Blast Summary (of sequences between 3 and 0 years older if possible)					
Allele/Clade	Accession	IsolateName	Host	Subtype	Region	Year	AvianAncestor	Probability	#BLAST	Host	HostType	Subtype	Region	Year
Avian A	M80968	A/swine/China/8/1978(H3N2)	Swine	H3N2	EasternAsia	1978	Dom-ans	0.46	13	Avian:100%	Dom-ans:69%	H6N1:15%	EasternAsia:77%	1977:1976-1978
Avian A (magenta on lilac)	CY037902	A/swine/Belgium/WVL1/1979(H1N1)	Swine	H1N1	WesternEurope	1979	Dom-ans	0.89	30	Avian:97%	Dom-ans:57%	H1N9:20%	EasternAsia:63%	1977:1976-1979
Avian A	GQ176140	A/mink/Sweden/3900/1984(H10N4)	Mink	H10N4	NorthernEurope	1984	Dom-ans	0.93	4	Avian:100%	Wild-ans:100%	H2N1:50%	WesternEurope:50%	1982:1981-1983
Avian B	M65020	A/equine/Jilin/1/1989(H3N8)	Equine	H3N8	EasternAsia	1989	Dom-ans	0.98	2	Avian:100%	Dom-ans:50%	H5N2:50%	EasternAsia:50%	1986:1986-1987
Avian A	CY085013	A/swine/Hong Kong/644/1993(H1N1)	Swine	H1N1	EasternAsia	1993	Dom-ans	0.33	4	Avian:50%	Dom-ans:50%	H1N1:50%	EasternAsia:100%	1992:1992-1993
Avian A	GU053113	A/England/AV877/1996(H7N7)	Human	H7N7	NorthernEurope	1996	Dom	manual	3	Avian:100%	Dom-ans:67%	H4N4:33%	EasternAsia:67%	1993:1993-1994
Avian A	CY115892	A/swine/Eire/89/1996(H1N1)	Swine	H1N1	NorthernEurope	1996	Dom-ans	0.66	2	Avian:50%	Dom-ans:50%	H7N4:50%	EasternAsia:50%	1994:1993-1996
Avian A (red/peach on yellow)	AF084285	A/Hong Kong/482/97(H5N1)	Human	H5N1	EasternAsia	1997	Dom-gal	manual	21	Avian:52%	NonAvian:48%	H5N1:81%	EasternAsia:100%	1997:1997-1997
Avian A	CY073456	A/swine/KU/2/2001(H11N6)	Swine	H11N6	EasternAsia	2001	Dom	no close	5	Avian:80%	Dom-ans:40%	H9N2:40%	EasternAsia:60%	2000:1998-2001
Avian B	CY067690	A/swine/KU/16/2001(H7N2)	Swine	H7N2	EasternAsia	2001	Dom-ans	0.98	11	Avian:100%	Wild-ans:55%	H6N2:18%	WesternEurope:55%	2000:1999-2001
Avian A (red on pink)	EF587281	A/Beijing/01/2003(H5N1)	Human	H5N1	EasternAsia	2003	Dom-ans	0.61	108	Avian:96%	Dom-gal:58%	H5N1:100%	EasternAsia:93%	2002:2000-2003
Avian A	AY342423	A/Netherlands/033/03(H7N7)	Human	H7N7	WesternEurope	2003	Dom-gal	1.00	25	Avian:96%	Wild-ans:60%	H7N7:20%	NorthernEurope:60%	2002:2000-2003
Avian B	EU587374	A/New_York/107/2003(H7N2)	Human	H7N2	NorthernAmerica	2003	Dom-gal	0.83	6	Avian:100%	Dom-gal:100%	H7N2:100%	NorthernAmerica:100%	2002:2001-2003
Avian A	CY015010	A/Canada/rv504/2004(H7N3)	Human	H7N3	NorthernAmerica	2004	Dom-gal	1.00	12	Avian:100%	Wild-ans:67%	H7N3:33%	NorthernAmerica:100%	2003:2001-2004
Avian A	AY790309	A/swine/Korea/S452/2004(H9N2)	Swine	H9N2	EasternAsia	2004	Dom-gal	manual	19	Avian:95%	Dom-gal:37%	H9N2:42%	EasternAsia:47%	2003:2001-2004
Avian A	JX500447	A/swine/Hubei/10/2008(H10N5)	Swine	H10N5	EasternAsia	2008	Dom-ans	0.72	13	Avian:100%	Wild-ans:69%	H4N6:23%	EasternAsia:77%	2007:2005-2008
Avian A	JQ815882	A/swine/Yangzhou/080/2009(H6N6)	Swine	H6N6	EasternAsia	2009	Dom-ans	0.99	45	Avian:100%	Dom-ans:98%	H6N6:58%	EasternAsia:100%	2007:2006-2009
Avian A	JX151011	A/swine/Guangdong/K4/2011(H4N8)	Swine	H4N8	EasternAsia	2011	Dom-ans	0.49	33	Avian:94%	Wild-ans:55%	H3N2:21%	EasternAsia:88%	2010:2008-2011
Avian A	CY125732	A/Mexico/InDRE7218/2012(H7N3)	Human	H7N3	CentralAmerica	2012	Dom	manual	7	Avian:100%	Wild-ans:71%	H10N7:29%	NorthernAmerica:71%	2011:2010-2012
Avian A (blue within yellow)	KJ406559	A/Jiangxi/IPB13/2013(H10N8)	Human	H10N8	EasternAsia	2013	Dom-gal	0.72	31	Avian:87%	Dom-gal:77%	H9N2:81%	EasternAsia:90%	2011:2010-2013
Avian A (purple within yellow)	KC896778	A/Nanjing/1/2013(H7N9)	Human	H7N9	EasternAsia	2013	Dom-gal	0.98	109	Avian:65%	Dom-gal:59%	H7N9:42%	EasternAsia:98%	2012:2010-2013

Wild Source							Ancestral State Estimation		Blast Summary (of sequences between 3 and 0 years older if possible)					
Allele	Accession	IsolateName	Host	Subtype	Region	Year	AvianAncestor	Probability	#BLAST	Host	HostType	Subtype	Region	Year
Avian A (brown on yellow)	CY028840	A/equine/Miami/1/1963(H3N8)	Equine	H3N8	NorthernAmerica	1963	Wild-ans	0.99	4	Equine:100%	NonAvian:100%	H3N8:100%	NorthernAmerica:50%	1963:1963-1963
Avian A	AB284067	A/seal/Massachusetts/1/1980(H7N7)	Seal	H7N7	NorthernAmerica	1980	Wild-ans	1.00	17	Avian:100%	Wild-ans:76%	H2N3:24%	NorthernAmerica:100%	1978:1977-1980
Avian A	M80947	A/seal/Massachusetts/133/1982(H4N5)	Seal	H4N5	NorthernAmerica	1982	Wild-ans	1.00	19	Avian:100%	Wild-ans:79%	H4N2:21%	NorthernAmerica:100%	1980:1979-1982
Avian A	KJ372724	A/whale/Maine/328B/1984(H13N2)	Whale	H13N2	NorthernAmerica	1984	Wild-other	0.98	5	Avian:100%	Wild-ans:60%	H13N6:40%	WesternEurope:60%	1983:1981-1983
Avian A	GU052287	A/seal/Massachusetts/3911/1992(H3N3)	Seal	H3N3	NorthernAmerica	1992	Wild-ans	0.98	86	Avian:100%	Wild-ans:88%	H11N9:13%	NorthernAmerica:100%	1980:1974-1990
Avian A	AF285889	A/swine/Ontario/01911-1/99(H4N6)	Swine	H4N6	NorthernAmerica	1999	Wild-ans	1.00	7	Avian:100%	Wild-ans:100%	H6N8:29%	NorthernAmerica:100%	1998:1996-1999
Avian B	GU052363	A/muskrat/Buryatiya/1944/2000(H4N6)	Muskrat	H4N6	EasternEurope	2000	Wild	manual	10	Avian:100%	Dom-gal:30%	H4N6:30%	EasternEurope:40%	1999:1998-2000
Avian A	AY619965	A/swine/Ontario/K01477/01(H3N3)	Swine	H3N3	NorthernAmerica	2001	Wild-ans	0.92	9	Avian:100%	Wild-ans:100%	H4N6:22%	NorthernAmerica:100%	1999:1998-1999
Avian B	AY619957	A/swine/Saskatchewan/18789/02(H1N1)	Swine	H1N1	NorthernAmerica	2002	Wild-ans	1.00	31	Avian:100%	Wild-ans:77%	H2N1:16%	NorthernAmerica:100%	2001:1999-2002
Avian A	KJ847690	A/Caspian_seal/Russia/1884/2002(H4N6)	Seal	H4N6	EasternEurope	2002	Wild-ans	0.96	10	Avian:100%	Wild-ans:80%	H10N9:20%	NorthernEurope:60%	2002:2001-2002
Avian A (green on green)	GU433352	A/canine/Guangdong/1/2006(H3N2)	Canine	H3N2	EasternAsia	2006	Wild-ans	0.50	32	Avian:97%	Wild-ans:91%	H4N6:38%	NorthernEurope:72%	2005:2003-2006
Avian B	FJ461601	A/swine/Korea/C13/2008(H5N2)	Swine	H5N2	EasternAsia	2008	Wild-ans	0.92	35	Avian:97%	Wild-ans:63%	H5N2:40%	EasternAsia:91%	2006:2005-2008
Avian A	GU215038	A/swine/Jilin/37/2008(H3N2)	Swine	H3N2	EasternAsia	2008	Wild-ans	0.49	24	Avian:100%	Wild-ans:62%	H4N6:29%	EasternAsia:71%	2007:2005-2008
Avian A	JX878679	A/swine/HuBei/06/2009(H4N1)	Swine	H4N1	EasternAsia	2009	Wild-ans	0.96	32	Avian:100%	Wild-ans:53%	H4N6:16%	EasternAsia:66%	2008:2006-2009
Avian A	JQ433883	A/harbor_seal/Massachusetts/1/2011(H3N8)	Seal	H3N8	NorthernAmerica	2011	Wild-ans	1.00	35	Avian:100%	Wild-ans:100%	H4N6:29%	NorthernAmerica:94%	2010:2008-2011

Undetermined							Ancestral State Estimation		Blast Summary (of sequences between 3 and 0 years older if possible)					
Allele	Accession	IsolateName	Host	Subtype	Region	Year	AvianAncestor	Probability	#BLAST	Host	HostType	Subtype	Region	Year
Avian A	AF333238	A/Brevig_Mission/1/1918(H1N1)	Human	H1N1	NorthernAmerica	1918	Avian	no close	39	Human:56%	NonAvian:56%	H1N1:59%	NorthernAmerica:67%	1957:1933-2007
Avian A (brown)	CY087820	A/equine/Prague/2/1956(H7N7)	Equine	H7N7	EasternEurope	1956	Avian	no close	2	Equine:100%	NonAvian:100%	H7N7:100%	EasternEurope:100%	1956:1956-1956

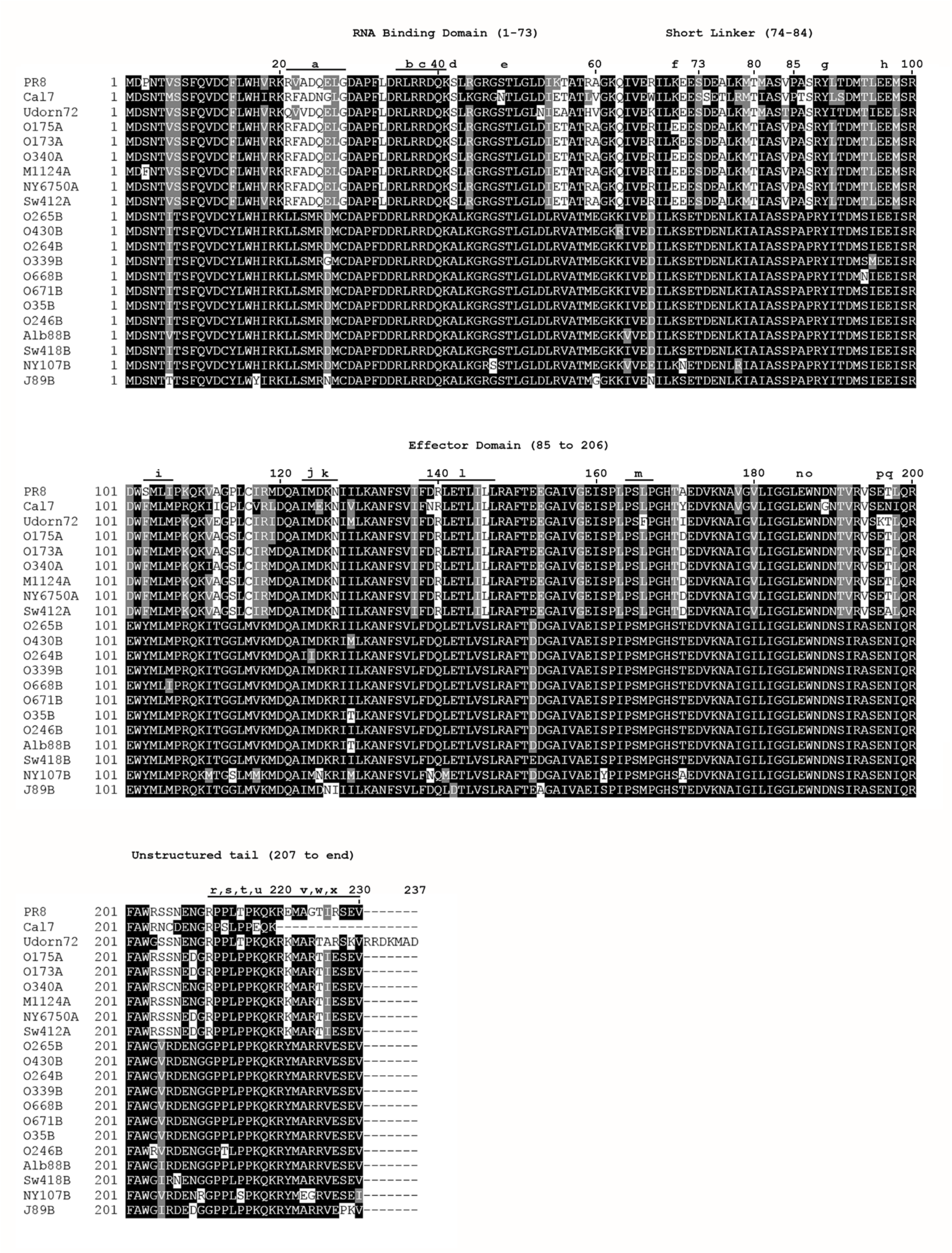

Fig S2. NS1 protein alignment. NS1 protein sequences from selected IAV strains were aligned using clustal omega (http://www.ebi.ac.uk/Tools/msa/clustalo/) using

default parameters. Alignments were processed using Boxshade Server (http://www.ch.embnet.org/software/BOX_form.html).

a – ^{21}RFADQELG^{28} and ^{21}LLSMRDMC^{28} conserved within A- and B- alleles, respectively (1).

b – 35-41 nuclear localisation signal (2-4).

c – R38 essential for RNA binding. K41 plays important role. (5).

d – S42 is a phosphorylation site (6).

e – S48 and T49 are phosphorylation sites (7). T49 phosphorylation inhibits IFN-antagonism (8).

f – K70 SUMOylation site (9).

g – Y89 mediates PI3K binding (10, 11).

h – E96 and E97 mediate TRIM25 binding and inhibition (12).

i – F103 and M106 required for CPSF30 binding and inhibition (13, 14).

j – 123-127 involved in PKR binding and inhibition (15).

k – D125G mutation (GAT > GTT) forms splice acceptor site and NS3 expression (16).

l – 138-147 nuclear export signal (17).

m – P164 and P167 involved in PI3K binding (10, 18).

n – E186 involved in CPSF30 binding and inhibition (19-21).

o – W187 required for effector domain dimerisation (22).

p – E196K mutation fails to inhibit IRF3 activation (23).

q - T197 phosphorylation site (7).

r - ^{211}GPPLPPKQKRYMARRV^{226} A2 rabbit polyclonal antiserum epitope.

s – ^{212}PPLPPK CRKL^{217} motif binds CRKI/II and CRKL proteins to prevent strong activation of JNK-AFT2 pathway (24, 25).

t – T215 phosphorylation site (25).

u – 216-229 second nuclear localisation signal (2-4).

v – K219 and K221 are SUMOylation sites (9, 26).

w – 223-230 involved in PABPII binding and inhibition (20, 27).

x – ^{227}ESEV^{230} motif for PDZ-domain binding and virulence factor (28, 29).

References

1. **Munir M, Zohari S, Metreveli G, Baule C, Belak S, Berg M.** 2011. Alleles A and B of non-structural protein 1 of avian influenza A viruses differentially inhibit beta interferon production in human and mink lung cells. The Journal of general virology **92:**2111-2121.
2. **Greenspan D, Palese P, Krystal M.** 1988. Two nuclear location signals in the influenza virus NS1 nonstructural protein. Journal of virology **62:**3020-3026.
3. **Han H, Cui ZQ, Wang W, Zhang ZP, Wei HP, Zhou YF, Zhang XE.** 2010. New regulatory mechanisms for the intracellular localization and trafficking of influenza A virus NS1 protein revealed by comparative analysis of A/PR/8/34 and A/Sydney/5/97. The Journal of general virology **91:**2907-2917.
4. **Melen K, Kinnunen L, Fagerlund R, Ikonen N, Twu KY, Krug RM, Julkunen I.** 2007. Nuclear and nucleolar targeting of influenza A virus NS1 protein: striking differences between different virus subtypes. Journal of virology **81:**5995-6006.
5. **Wang W, Riedel K, Lynch P, Chien CY, Montelione GT, Krug RM.** 1999. RNA binding by the novel helical domain of the influenza virus NS1 protein requires its dimer structure and a small number of specific basic amino acids. Rna **5:**195-205.
6. **Hsiang TY, Zhou L, Krug RM.** 2012. Roles of the phosphorylation of specific serines and threonines in the NS1 protein of human influenza A viruses. Journal of virology **86:**10370-10376.
7. **Hutchinson EC, Denham EM, Thomas B, Trudgian DC, Hester SS, Ridlova G, York A, Turrell L, Fodor E.** 2012. Mapping the phosphoproteome of influenza A and B viruses by mass spectrometry. PLoS pathogens **8:**e1002993.
8. **Kathum OA, Schrader T, Anhlan D, Nordhoff C, Liedmann S, Pande A, Mellmann A, Ehrhardt C, Wixler V, Ludwig S.** 2016. Phosphorylation of influenza A virus NS1 protein at threonine 49 suppresses its interferon antagonistic activity. Cellular microbiology **18:**784-791.
9. **Santos A, Pal S, Chacon J, Meraz K, Gonzalez J, Prieto K, Rosas-Acosta G.** 2013. SUMOylation Affects the Interferon Blocking Activity of the Influenza A Nonstructural Protein NS1 without Affecting Its Stability or Cellular Localization. Journal of virology **87:**5602-5620.
10. **Hale BG, Kerry PS, Jackson D, Precious BL, Gray A, Killip MJ, Randall RE, Russell RJ.** 2010. Structural insights into phosphoinositide 3-kinase activation by the influenza A virus NS1 protein. Proceedings of the National Academy of Sciences of the United States of America **107:**1954-1959.
11. **Hale BG, Jackson D, Chen YH, Lamb RA, Randall RE.** 2006. Influenza A virus NS1 protein binds p85beta and activates phosphatidylinositol-3-kinase signaling.

Proceedings of the National Academy of Sciences of the United States of America **103:**14194-14199.
12. **Gack MU, Albrecht RA, Urano T, Inn KS, Huang IC, Carnero E, Farzan M, Inoue S, Jung JU, Garcia-Sastre A.** 2009. Influenza A virus NS1 targets the ubiquitin ligase TRIM25 to evade recognition by the host viral RNA sensor RIG-I. Cell host & microbe **5:**439-449.
13. **Kochs G, Garcia-Sastre A, Martinez-Sobrido L.** 2007. Multiple anti-interferon actions of the influenza A virus NS1 protein. Journal of virology **81:**7011-7021.
14. **Das K, Ma LC, Xiao R, Radvansky B, Aramini J, Zhao L, Marklund J, Kuo RL, Twu KY, Arnold E, Krug RM, Montelione GT.** 2008. Structural basis for suppression of a host antiviral response by influenza A virus. Proceedings of the National Academy of Sciences of the United States of America **105:**13093-13098.
15. **Min JY, Li S, Sen GC, Krug RM.** 2007. A site on the influenza A virus NS1 protein mediates both inhibition of PKR activation and temporal regulation of viral RNA synthesis. Virology **363:**236-243.
16. **Selman M, Dankar SK, Forbes NE, Jia J-J, Brown EG.** 2012. Adaptive mutation in influenza A virus non-structural gene is linked to host switching and induces a novel protein by alternative splicing. Emerging Microbes & Infections **1:**e42.
17. **Li Y, Yamakita Y, Krug RM.** 1998. Regulation of a nuclear export signal by an adjacent inhibitory sequence: the effector domain of the influenza virus NS1 protein. Proceedings of the National Academy of Sciences of the United States of America **95:**4864-4869.
18. **Shin YK, Li Y, Liu Q, Anderson DH, Babiuk LA, Zhou Y.** 2007. SH3 binding motif 1 in influenza A virus NS1 protein is essential for PI3K/Akt signaling pathway activation. Journal of virology **81:**12730-12739.
19. **Nemeroff ME, Barabino SML, Li Y, Keller W, Krug RM.** 1998. Influenza Virus NS1 Protein Interacts with the Cellular 30kDa Subunit of CPSF and Inhibits 3' End Formation of Cellular Pre-mRNAs. Molecular Cell **1:**991-1000.
20. **Li Y, Chen ZY, Wang W, Baker CC, Krug RM.** 2001. The 3'-end-processing factor CPSF is required for the splicing of single-intron pre-mRNAs in vivo. Rna **7:**920-931.
21. **Noah DL, Twu KY, Krug RM.** 2003. Cellular antiviral responses against influenza A virus are countered at the posttranscriptional level by the viral NS1A protein via its binding to a cellular protein required for the 3' end processing of cellular pre-mRNAS. Virology **307:**386-395.
22. **Kerry PS, Long E, Taylor MA, Russell RJ.** 2011. Conservation of a crystallographic interface suggests a role for beta-sheet augmentation in influenza virus NS1 multifunctionality. Acta crystallographica. Section F, Structural biology and crystallization communications **67:**858-861.
23. **Kuo RL, Zhao C, Malur M, Krug RM.** 2010. Influenza A virus strains that circulate in humans differ in the ability of their NS1 proteins to block the activation of IRF3 and interferon-beta transcription. Virology **408:**146-158.
24. **Heikkinen LS, Kazlauskas A, Melen K, Wagner R, Ziegler T, Julkunen I, Saksela K.** 2008. Avian and 1918 Spanish influenza a virus NS1 proteins bind to Crk/CrkL Src homology 3 domains to activate host cell signaling. The Journal of biological chemistry **283:**5719-5727.
25. **Hale BG, Knebel A, Botting CH, Galloway CS, Precious BL, Jackson D, Elliott RM, Randall RE.** 2009. CDK/ERK-mediated phosphorylation of the human influenza A virus NS1 protein at threonine-215. Virology **383:**6-11.

26. **Xu K, Klenk C, Liu B, Keiner B, Cheng J, Zheng BJ, Li L, Han Q, Wang C, Li T, Chen Z, Shu Y, Liu J, Klenk HD, Sun B.** 2011. Modification of nonstructural protein 1 of influenza A virus by SUMO1. Journal of virology **85:**1086-1098.
27. **Chen Z, Li Y, Krug RM.** 1999. Influenza A virus NS1 protein targets poly(A)-binding protein II of the cellular 3'-end processing machinery. The EMBO Journal **18:**2273-2283.
28. **Obenauer JC, Denson J, Mehta PK, Su X, Mukatira S, Finkelstein DB, Xu X, Wang J, Ma J, Fan Y, Rakestraw KM, Webster RG, Hoffmann E, Krauss S, Zheng J, Zhang Z, Naeve CW.** 2006. Large-scale sequence analysis of avian influenza isolates. Science **311:**1576-1580.
29. **Jackson D, Hossain MJ, Hickman D, Perez DR, Lamb RA.** 2008. A new influenza virus virulence determinant: the NS1 protein four C-terminal residues modulate pathogenicity. Proceedings of the National Academy of Sciences of the United States of America **105:**4381-4386.
